# Compounded effects on wetland greenhouse gas fluxes from climate change and water management along a saline to freshwater gradient

**DOI:** 10.1073/pnas.2513685123

**Published:** 2026-02-17

**Authors:** Cheryl L. Doughty, Qing Ying, Eric Ward, Erin Delaria, Glenn M. Wolfe, Sparkle L. Malone, David E. Reed, Tiffany Troxler, John S. Kominoski, Edward Castañeda-Moya, David Yannick, Gregory Starr, Steven F. Oberbauer, Abigail Barenblitt, Anthony Campbell, Sean Charles, Lola Fatoyinbo, Jonathan Gewirtzman, Thomas Hanisco, Reem Hannun, Stephan Kawa, David Lagomasino, Leslie Lait, Ayia Lindquist, Paul Newman, Peter Raymond, Judith Rosentreter, Kenneth Thornhill, Derrick Vaughn, Benjamin Poulter

**Affiliations:** ^a^Earth System Science Interdisciplinary Center, University of Maryland, College Park, MD 20740; ^b^Biospheric Sciences Laboratory, National Aeronautics and Space Administration Goddard Space Flight Center, Greenbelt, MD 20771; ^c^Atmospheric Chemistry and Dynamics Laboratory, National Aeronautics and Space Administration Goddard Space Flight Center, Greenbelt, MD 20771; ^d^Yale School of the Environment, Yale University, New Haven, CT 06511; ^e^Institute of Environment, Florida International University, Miami, FL 33199; ^f^Department of Earth and Environment, Florida International University, Miami, FL 33199; ^g^Department of Biological Sciences, Florida International University, Miami, FL 33199; ^h^Department of Biological Sciences, University of Alabama, Tuscaloosa, AL 35487; ^i^Department of Geographical Sciences, University of Maryland, College Park, MD 20740; ^j^Goddard Earth Sciences Technology and Research II, University of Maryland Baltimore County, Baltimore, MD 21250; ^k^Integrated Coastal Program, East Carolina University, Outer Banks, NC 27981; ^l^Atmospheric Science Branch, National Aeronautics and Space Administration Ames Research Center, Moffett Field, CA 94035; ^m^Earth Sciences Division, National Aeronautics and Space Administration Goddard Space Flight Center, Greenbelt, MD 20771; ^n^Faculty of Science and Engineering, Southern Cross University, East Lismore, NSW 2480, Australia; ^o^National Aeronautics and Space Administration Langley Research Center, Hampton, VA 23666; ^p^Department of Geosciences, Utah State University, Logan, UT 84322

**Keywords:** carbon monitoring system, upscale model, blue carbon, teal carbon, methane

## Abstract

Saline and freshwater wetlands store large amounts of carbon, which has driven interest in their role as nature-based climate solutions. Because these ecosystems can be both sinks and sources of carbon to the atmosphere as environmental conditions and human influence change, the net climate mitigation potential of wetlands at regional to global scales remains uncertain. We used a data-driven approach to measure ground-based and airborne fluxes to upscale carbon dioxide (CO_2_) and methane (CH_4_) fluxes using satellite-based surface reflectances at 500-m resolution across a gradient of saline to freshwater wetlands in Southern Florida, USA. Daily time series of CO_2_ and CH_4_ fluxes from 2000 to 2024 integrated surface properties related to vegetation productivity, flooding, and disturbance, and captured 80% and 91% of the variability in annual fluxes of CO_2_ and CH_4_, respectively. Long-term (23-y) patterns in the fluxes of CH_4_, CO_2_, and their CO_2_-equivalent (CO_2_eq) are represented as Global Warming Potential 100 (GWP100) and were shown to vary spatially with wetland management, revealing higher carbon uptake in mangroves susceptible to hurricane damage and coastal hydrology, and greater carbon emissions in freshwater sawgrass marshes where freshwater hydrology is managed for restoration. Regional net annual CO_2_eq uptake in coastal and freshwater wetlands increased by 18% from −7.0 ± 3.3 MMT CO_2_eq y^−1^ in ~2003 to −8.4 ± 3.8 MMT CO_2_eq y^−1^ in ~2020 at an uptake rate of −0.06 ± 0.01 MMT CO_2_eq y^−2^. Annually, roughly 43% of CO_2_ uptake was offset by CH_4_ emissions from all wetlands in the region (from 16% in mangroves to 82% in freshwater marshes).

## Significance

To manage a large wetland landscape like the Everglades as a net carbon sink, carbon uptake and emissions must be balanced along a gradient of coastal saline mangroves and marshes to nontidal freshwater marshes and forests. Pairing ground and airborne measurements with long-term satellite imagery helps monitor how greenhouse gas exchange changes with wetland vegetation, salt and freshwater levels, disturbances, and management of these compounding factors. Our dataset revealed the importance of restoring hydrologic flows to potentially increase aerobic conditions that minimize freshwater marshes as methane sources and to maximize carbon dioxide uptake in healthy and recovering mangroves. Data upscaling enabled a landscape perspective of carbon exchange needed to improve carbon inventories and manage diverse wetlands as nature-based climate solutions.

Wetlands store large amounts of carbon in their soils, which has driven interest in their role as nature-based climate solutions. In recent years, tidal wetlands such as mangroves and saltmarshes have garnered attention as “blue carbon” ecosystems where carbon (C) is both fixed as plant biomass and trapped from marine, estuarine, or riverine sources, then stored in soils for centuries to millennia (1–3). To a lesser extent, nontidal freshwater wetlands, such as marshes, swamps, and peatlands, have also been noted as “teal” carbon ecosystems for a similarly high capacity to trap and store C in inundated soils (4, 5), though with potentially higher emissions of methane (6). Both tidal and nontidal wetlands thus have high potential as natural solutions to offset global C emissions if properly protected and managed (7–11), however, optimizing the full potential of wetlands with certainty remains complicated by high spatial and temporal variability in the uptake and emissions of greenhouse gases (GHGs). This variability is driven by differences in wetland vegetation, salinity, and hydrological conditions across wetland complexes, especially those in coastal regions (12, 13). Understanding whether wetlands can serve as natural climate solutions will require comprehensive monitoring across diverse ecosystems and robust estimates of uncertainty over space and time (14–16).

Many approaches to understand regional, national, or global GHG emissions and removals from wetlands often rely on globally averaged estimates of stored C, emission factors, and areal extents (17, 18). How GHG emissions and uptake from wetlands will respond to a changing environment requires more spatial information, and a better understanding of the temporal variation in emissions makes GHG accounting more accurate (19). That is because the exchange of carbon dioxide (CO_2_) and methane (CH_4_) GHGs in wetlands is as inherently variable as the ecogeomorphic interactions between wetland plants and environmental gradients that mediate C uptake and burial processes (20, 21). Environmental change and human disturbance further alter the drivers and processes controlling C dynamics across many diverse wetland types (13, 22–26). As wetlands face the compounded effects of climate change and direct human disturbance, their ability to sequester and store C will be differentially altered by increasing sea levels, temperatures, and extreme weather events like hurricanes, as well as the adaptive management of hydrology, sediment, and land cover changes (25, 27, 28).

**Fig. 1.**
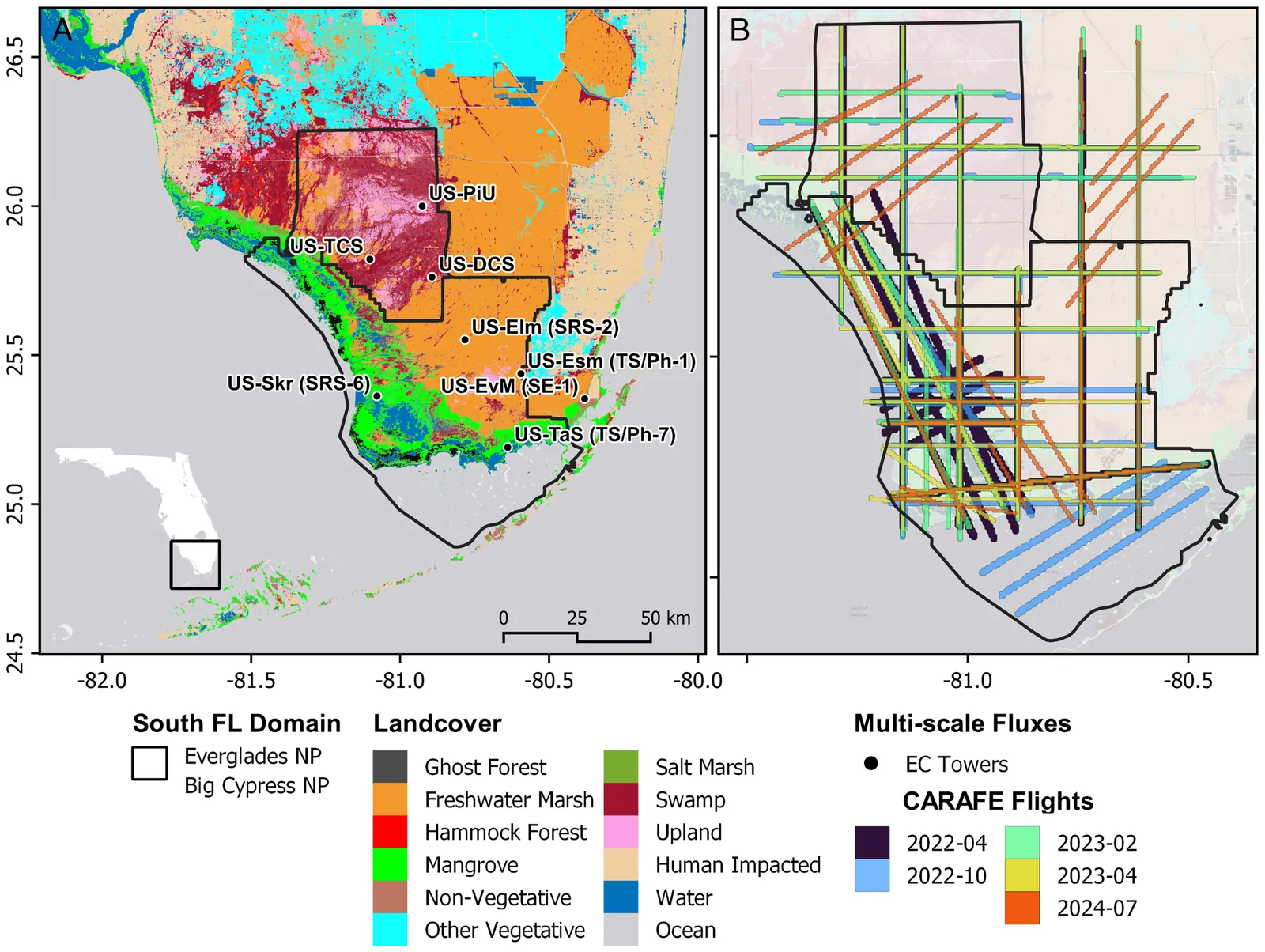
The BlueFlux study domain over the tidal to freshwater wetlands of the Everglades where multiscale EC flux observations were collected from (*A*) AmeriFlux towers (with corresponding FCE LTER tower name) and (*B*) airborne CARAFE flight deployments over the ENP and BCNP of South Florida, USA.

Nowhere are the compounding effects of human influence and environmental change more evident than in the modern-day Everglades. This expansive network of oceanic, coastal, and terrestrial ecosystems have been largely shaped by environmental changes in sea levels over millennia and more recently, by human needs to redistribute water in support of urban and agricultural development (29). Landscape-scale management efforts are currently underway to restore water flows in support of healthy Everglades ecosystems (30). The timing and amount of freshwater is controlled as it flows south from Lake Okeechobee into two main drainages, the Shark River Slough and Taylor Slough/Panhandle, which each converge with varying tidal influence to form a dynamic coastal-freshwater gradient (31). The movement of salinity and nutrients across this landscape are evident as wetland vegetation transitions from coastal, marine mangroves to more brackish saltmarshes and swamps, and inland to freshwater wetlands where small topographical differences create a mosaic of freshwater marsh sloughs, tree islands, forested cypress swamps, and hardwood hammocks characterized by different hydroperiod, or flooding duration (Fig. 1, 32). These diverse wetlands have been the focus of decades of long-term carbon-cycling research across environmental (i.e., hydrology, salinity, nutrient) and regulatory (i.e., water management, restoration, landuse) gradients (29). Ground-based studies on the variable impacts of freshwater supply, salinity, and temperature driving biogeochemical processes have revealed mangroves as net carbon sinks due to high primary production, whereas freshwater marshes were both net sinks and sources with varying hydroperiod (31). Yet, net C budgets at the landscape-scale would benefit from cross-system studies that factor methane in C cycling.

We present an approach using a combination of ground-based and novel airborne eddy flux measurements paired with a time series of satellite-based surface reflectance for data-driven upscaling of daily CO_2_ and CH_4_ fluxes for over 20 y in the Everglades region (Florida, USA). This study is part of the ongoing NASA BlueFlux effort to better understand the variability and drivers of fluxes in the complex mosaic of wetlands and adjacent uplands in South Florida (33). Our ensemble random forest models predicted ecosystem CO_2_ and CH_4_ fluxes at a daily, 500-m scale from February 2000 to August 2024. We identify the high spatial and temporal heterogeneity of long-term patterns in CH_4_, CO_2_, and net CO_2_-equivalent (CO_2_eq, GWP100) fluxes arising from differences in environmental disturbances due to hydrology, hurricanes, and fires in relation to the management of wetland habitats across the saline-freshwater ecotone of the Everglades. Our C monitoring products provide the high spatiotemporal resolution information on C dynamics that are essential for the management of tidal to freshwater wetlands facing environmental stressors and human impacts.

## Results and Discussion

### Airborne and Ground Observations Capture Heterogeneous Wetland Fluxes.

Airborne and ground-based eddy covariance (EC) fluxes of CO_2_ and CH_4_ provide complementary measures of the variability of ecosystem fluxes across space and time (Fig. 1). The NASA Carbon Airborne Flux Experiment (CARAFE) instrument was designed to bridge gaps in top–down and bottom–up approaches by quantifying surface fluxes from local (<1 km) to regional (>1,000 km) scales (34), where insights are needed to resolve spatial gradients in landscape-scale fluxes (35), and address regional data gaps in wetland carbon fluxes (36). We deployed CARAFE over the heterogeneous wetlands of Southern Florida during wet and dry seasons from 2022 to 2024 and detected differing seasonal rates of CO_2_ and CH_4_ fluxes among the dominant mangrove, swamp, saltmarsh, and freshwater marsh vegetation classes found across the Everglades (37).

The regional tower network in South Florida was an invaluable source of data collected since 2004 on the vertical net ecosystem exchange (NEE) of land-atmosphere C exchange (31, 38, 39) from eight EC towers representing varying hydrology and disturbance histories among riverine, scrub, and encroaching mangroves (40–43), saltmarsh (44), freshwater marsh (45–47), as well as dwarf and tall cypress swamps, and pine uplands (48, 49) (*SI Appendix*, Table S1 and *Extended Methods*). Half-hourly tower fluxes (*SI Appendix*, Table S1) and 1-Hz airborne CARAFE fluxes (50) were processed to match the spatial and daily temporal resolution of MODerate-resolution Imaging Spectroradiometer (MODIS) by averaging samples within a 500-m grid and estimating daily integrated averages of CO_2_ and CH_4_ fluxes (*Material and Methods*). We found that the additional sampling and broader spatial coverage provided by airborne EC improved prediction errors for daily CO_2_ by 22% across the heterogeneous landscape compared to towers alone. Towers, however, by providing repeated measures at fixed locations captured 18% and 37% more of the temporal variability in daily CH_4_ and CO_2_ fluxes (*SI Appendix*, Table S2). Together, multiscale observations from CARAFE and towers provided improved geographic and temporal coverage of fluxes by capturing more variability across wetland habitats, disturbances, and seasons in South Florida which increase the generalizability of upscaling models across a diverse and dynamic wetland gradient.

### Data-Driven Upscaling of Daily CO_2_ and CH_4_ Fluxes.

Our upscaling approach used MODIS time series of daily surface reflectance as the predictors in ensemble machine learning models to upscale fluxes observed from long-term ground monitoring and novel continuous aerial flux sampling (*Material and Methods*). Upscaling models predicted 65% of variance in daily CO_2_ flux (RMSE = 0.80 μmol m^−2^ s^−1^, MAE = 0.91 μmol m^−2^ s^−1^) and 47% of variance in daily CH_4_ (RMSE = 27.7 nmol m^−2^ s^−1^, MAE = 29.8 nmol m^−2^ s^−1^) as a function of seven surface reflectance bands (blue, green, red, NIR1, NIR2, SWIR1, and SWIR2). Ensemble modeling outputs include predicted 500-m gridded daily average CO_2_ flux ± SD (μmol m^−2^ s^−1^) and daily average CH_4_ flux ± SD (nmol m^−2^ s^−1^) dating from February 2000 to August 2024, available as the BlueFlux C monitoring prototypes (51). Other data-driven methane upscaling approaches predicted 59 to 64% of the variability in global, extratropical freshwater wetland CH_4_ flux (52) and 67% of daily CH_4_ fluxes in just the Southeast US (53). In northern high-latitude wetlands, methane upscaling from EC towers accurately predicted 51% of daily fluxes and 62% of weekly CH_4_ fluxes (54). At the daily scale, our upscaling demonstrates an inherently high level of spatial and temporal variability of atmosphere-ecosystem fluxes of CO_2_ and CH_4_ that is only evident with increased airborne observations and EC towers representing holistic vegetative communities over time and space.

**Fig. 2.**
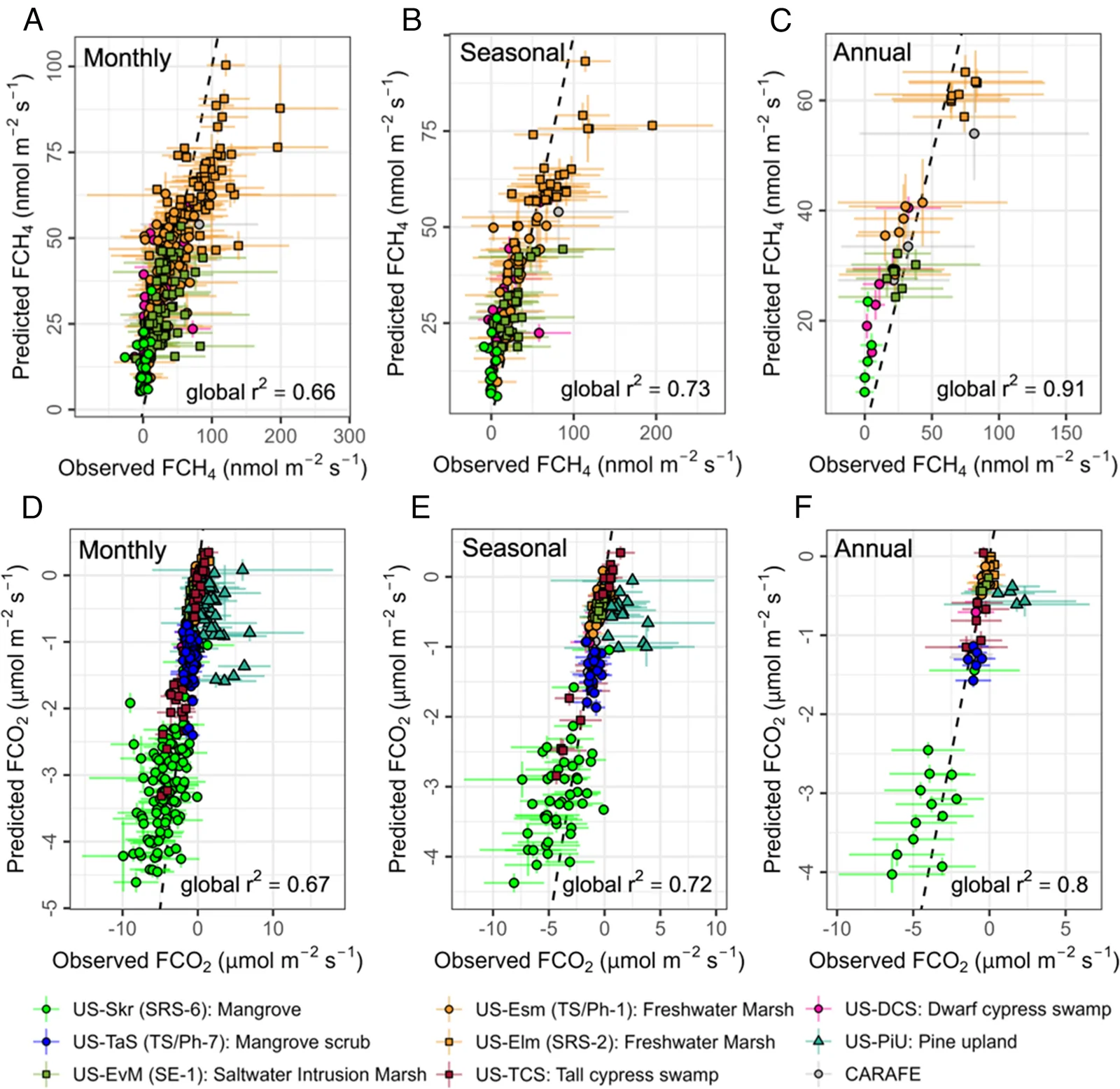
Modeled flux validation using ground and airborne observations for (*Top*) CH_4_ (*A*) monthly, (*B*) seasonal, and (*C*) annual flux (mean nmol m^−2^ s^−1^ ± SD), and (*Bottom*) CO_2_ (*D*) monthly, (*E*) seasonal, and (*F*) annual flux (mean flux ± SD μmol m^−2^ s^−1^). Error bars for each record represent the SD in flux measurements and in the ensemble predictions. Dotted lines indicate a 1:1 relationship.

The power to predict fluxes across the heterogeneous wetlands increased when aggregated to monthly, seasonal, and annual time scales (Fig. 2). Monthly fluxes accurately represented 67% of observed variability in monthly CO_2_ (RMSE = 0.65 μmol m^−2^ s^−1^) and 66% of observed CH_4_ (RMSE = 11.1 nmol m^−2^ s^−1^). Seasonal fluxes captured 72% and 73% of observed CO_2_ (RMSE = 0.60 μmol m^−2^ s^−1^) and CH_4_ (RMSE = 9.6 nmol m^−2^ s^−1^), respectively. At the annual scale, modeled fluxes explained 80% of variance in CO_2_ (RMSE = 0.49 μmol m^−2^ s^−1^) and 91% in CH_4_ (RMSE = 5.0 nmol m^−2^ s^−1^) fluxes compared to observed ground and airborne fluxes. There were clear differences among vegetation types at EC towers (Fig. 2 and *SI Appendix*, Table S3). Riverine mangrove towers had the highest annual CO_2_ uptake rates predicted among the other wetland vegetation types (r^2^= 0.50; *P* = 0.008). Conversely, freshwater marsh towers had higher annual CH_4_ emissions than any other wetland type (r^2^= 0.43; *P* = 0.1). Monthly, seasonal, and annual flux predictions increasingly captured the variance across diverse wetland landscapes by reducing the variability caused by short-term fluctuations while propagating robust estimates of the spatiotemporal variance at finer time scales.

### Regional Trends of CO_2_, CH_4_, CO_2_eq Reveal a Delicately Balanced Wetland Carbon Sink.

**Fig. 3.**
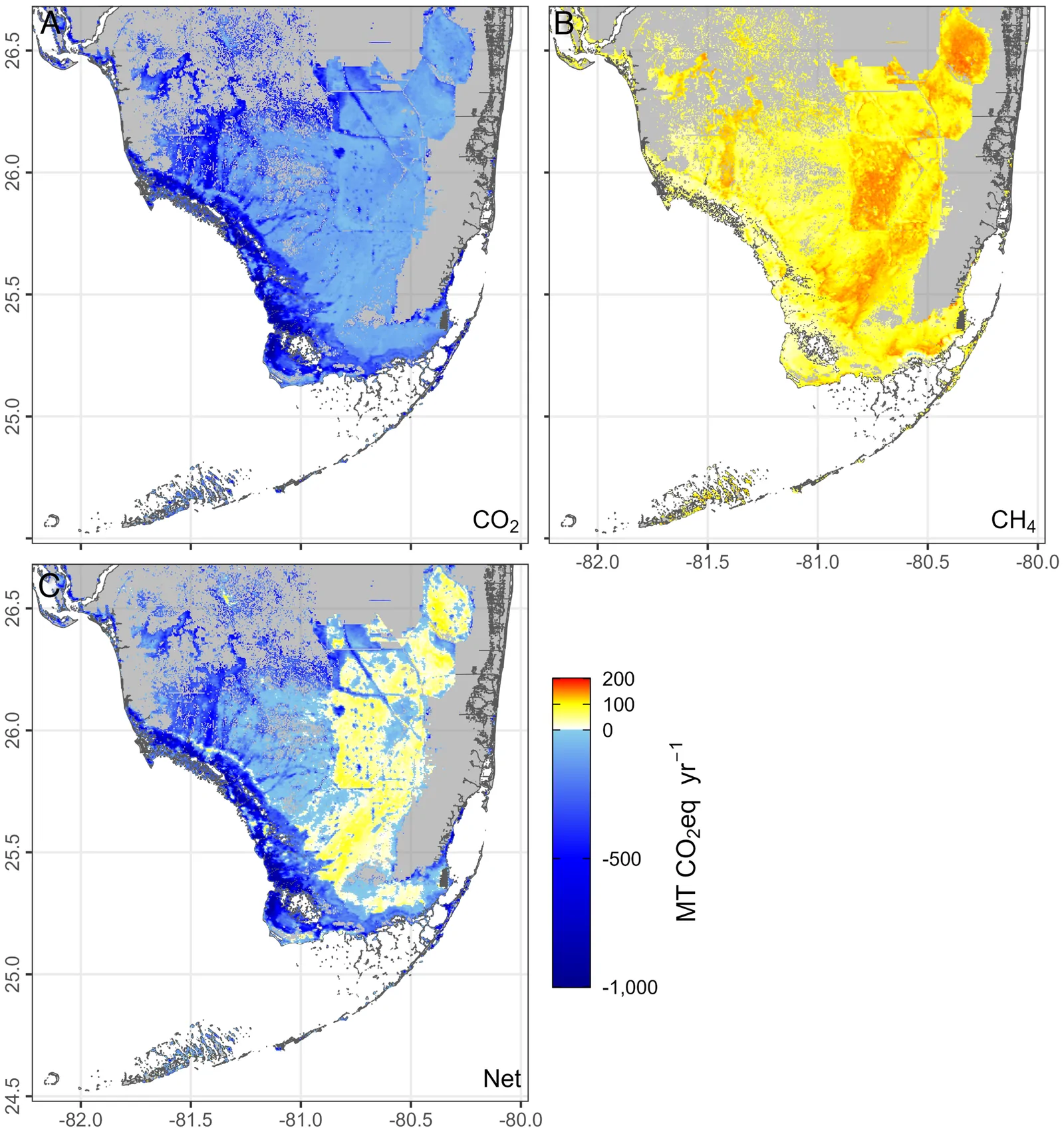
Predicted 5-y average (*A*) CO_2_, (*B*) CH_4_, and (*C*) net CO_2_eq flux (MT CO_2_eq y^−1^) from 2019 to 2023.

**Fig. 4.**
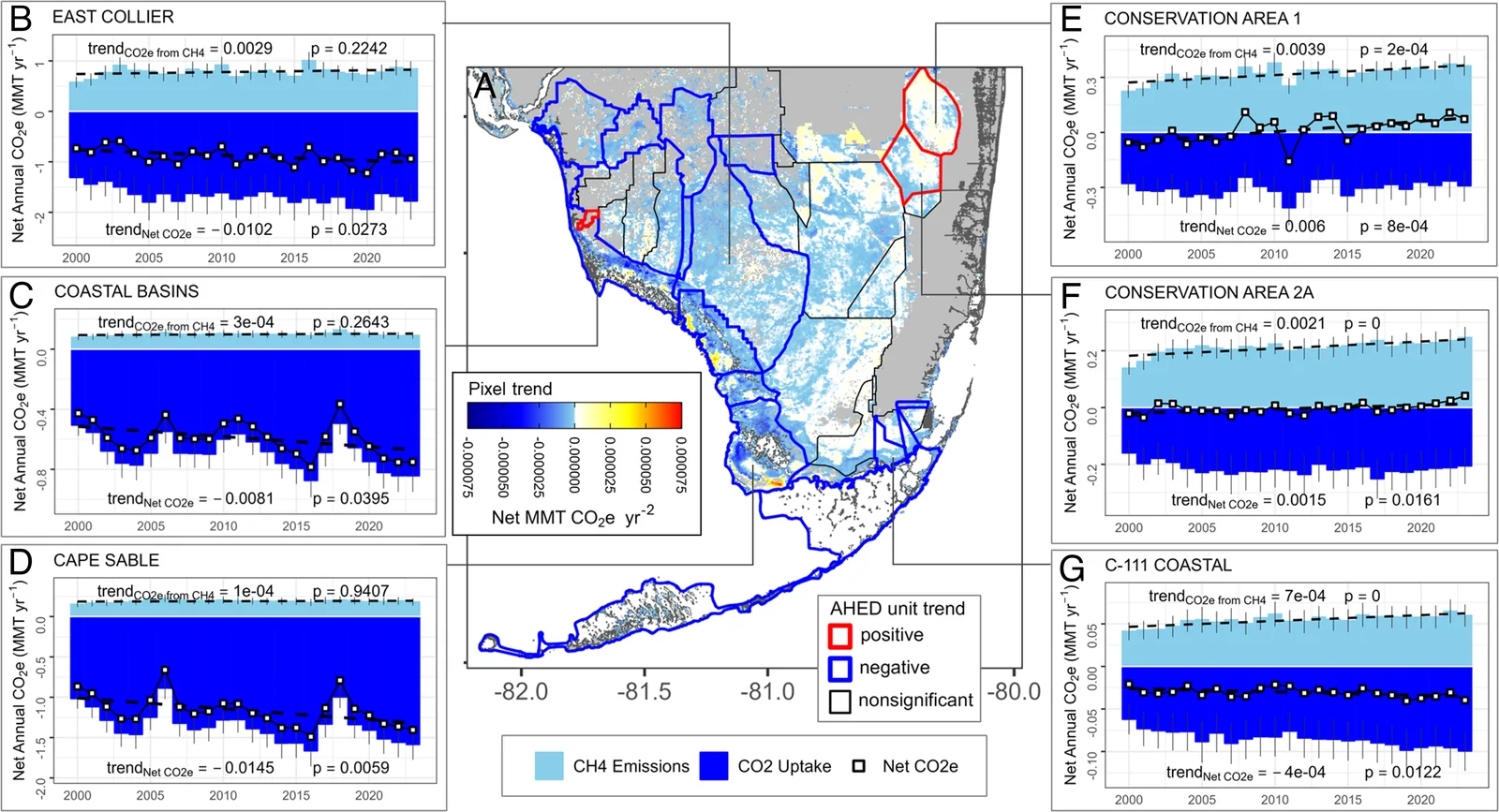
Trends in CO_2_eq from CO_2_ and CH_4_ fluxes. (*A*) Long-term (2000 to 2023) trends per pixel and per AHED water management unit. Annual time series of net CO_2_eq uptake and emissions from CO_2_ and CH_4_ fluxes for selected AHED units: (*B*) East Collier, (*C*) Coastal Basin, (*D*) Cape Sable, (*E*) Conservation Area 1, (*F*) Conservation Area 2A, and (*G*) C-111 Coastal. Error bars show the SD from ensemble predicted fluxes in CO_2_eq from CO_2_ and CH_4_ fluxes (MMT y^−1^). Explore results using an interactive web app: https://ee-cdoughty.projects.earthengine.app/view/blueflux-ghg-tracker.

Together, upscaled CO_2_ and CH_4_ fluxes captured over 23 y of regional CO_2_eq variability needed for robust GHG accounting (Figs. 3 and 4). We estimated that on average the annual net CO_2_ flux (excluding methane emissions) was −13.7 ± 1.11 MMT CO_2_eq y^−1^ (±SD), whereby a negative flux indicates CO_2_ uptake or removal from the atmosphere. The annual average net CH_4_ flux was 0.21 ± 0.02 Tg CH_4_ y^−1^ or 5.8 ± 0.5 MMT CO_2_eq y^−1^, which conversely, signifies the addition of CH_4_ emissions to the atmosphere. We calculated net CO_2_eq flux as the sum of CO_2_ and CH_4_ fluxes after correcting for the IPCC 100-y global warming potential (GWP) value of 27.0 for nonfossil methane (55). Less conservative GWP values may better account for changes in sustained emission rates over time (56–58).

Overall, we estimated a regional trend in net CO_2_eq (including CO_2_ and CH_4_) of −0.06 ± 0.01 MMT y^−2^ for all wetlands combined in the study domain (*P* = 0.02, *SI Appendix*, Fig. S1). The overall net CO_2_eq uptake from 2000 to 2023 was driven largely by a negative trend in CO_2_ uptake (−0.085 ± 0.007 MMT CO_2_eq y^−2^), and offset by a positive trend in CH_4_ emissions (0.036 ± 0.005 MMT CO_2_eq y^−2^). We estimated long-term trends in annual fluxes using Sen’s slope to calculate trend magnitude in nonparametric time series data (59). Net annual CO_2_eq uptake increased over the region by 18% from −7.0 ± 3.3 MMT CO_2_eq y^−1^ in ~2003 to −8.4 ± 3.8 MMT CO_2_eq y^−1^ in ~2020 based on 5-y averages (Fig. 3). However, the amount of regional uptake from year to year varied by as much as 24% (*SI Appendix*, Fig. S1). From 2000 to 2023, on average roughly 42.8 ± 2.8% of CO_2_ uptake was offset by CH_4_ emissions annually from all wetlands.

Offset ratios of CH_4_ emissions to CO_2_ uptake, however, also varied greatly among wetland vegetation, levels of protection, and hydrological management over time (*SI Appendix*, Figs. S2 and S3 and Tables S4 and S5). By focusing on areas where wetland vegetation has been consistent from 2000 to 2023, the long-term CH_4_ offset to CO_2_ uptake in CO_2_eq was found to be 82% in freshwater marsh, 33% in swamps, 30% in saltmarshes, and 16% in mangroves (*SI Appendix*, Table S4). The 5% offset we estimated with preliminary upscaling models using aircraft flux data only from April 2022 (33), was likely biased toward greater seasonal carbon uptake in tidal wetlands (37). Our methane offset in freshwater marshes regionally was estimated at 8% before converting to CO_2_eq (*SI Appendix*, Table S4), which is higher than the 5% offset estimated prior to restoration activities in a wet-season sawgrass marsh (60), where disturbances and seasonal differences occurring within and among wetland vegetation types may be underrepresented (61). Methane emissions may offset CO_2_ uptake by as much as 14% in saltmarshes, 36% in mangroves, and over 60% in nontidal wetlands globally (62). By including seasonally and spatially representative flux measurements from aircraft or towers, we gain a regional perspective of net carbon balance while maintaining robust estimates of the variability at 500-m needed to assess local patterns and drivers across very different wetlands.

### Net C Balance Varies Long-Term across Diverse and Managed Wetlands.

Our regional perspective of net C uptake highlights the underlying variability in fluxes across the region (Fig. 3) and allows us to contextualize important differences among the region’s protected areas, water management units, and diverse wetland types (Fig. 4 and *SI Appendix*, Figs. S2 and S3). We developed an interactive approach to explore trends and patterns in predicted annual CO_2_, CH_4_, and CO_2_eq fluxes across the data-rich region (GEE Web App Link).

Patterns in wetland fluxes of CO_2_eq over time varied among regionally important spatial units like the USGS (HUC10) watersheds that comprise the South Florida Water Management District’s (SFWMD) Arc Hydro Enhanced Database (AHED). Each watershed designates a water management unit as part of the Comprehensive Everglades Restoration Plan (CERP) that aims to restore and protect South Florida’s diverse ecosystems while balancing human-related needs for water supply and flood protection (63). Water management units contain wetland areas with similar vegetative communities (*SI Appendix*, Fig. S5), hydrological conditions, and active control of freshwater levels, contributing to highly variable uptake of CO_2_eq across the region (Fig. 4).

Water Conservation Areas 1 and 2A showcase where net CO_2_eq trends were positive and indicate both abrupt and gradual conversion of C sinks to sources over time due to disturbances related to hydrologic management and increased contributions of CH_4_ to annual net CO_2_eq emissions in predominantly freshwater marshes (Fig. 4 *E* and *F*). These units exemplify hotspots of high methane emissions found in freshwater marshes across the region (Fig. 3*B*), where the active control of water flow magnitude and direction has had long-term implications for surface water and vegetation health (*SI Appendix*, Fig. S5). Although observations over freshwater marsh from airborne EC accurately represented the habitat composition of the study region (*SI Appendix*, Fig. S4), future deployments will target more sampling of seasonal marsh disturbance with higher spatial resolution to connect with disturbance histories.

Several other water management units had significant negative trends in net CO_2_eq where increased CO_2_ uptake outweighed CH_4_ emissions over the study period. These correspond largely to coastal areas containing mangroves and forested swamps where woody plants mediate high rates of CO_2_ uptake (Fig. 3*A*), and saline waters containing sulfate reduce methane production (64, 65). Net CO_2_eq balance could change as interannual CO_2_ drawdown fluctuates (Fig. 4*B*), CH_4_ fluxes outpace CO_2_ fluxes (Fig. 4*G*), and environmental disturbances to specific tidal wetlands impact their capacity to sequester CO_2_ (Fig. 4 *C* and *D*).

The effects of major disturbances are evident as pulses of reduced predicted CO_2_ uptake rates in the Coastal Basins and Cape Sable regions (Fig. 4 *C* and *D*). We identified the same pattern over time in predicted CO_2_ uptake rates for all mangrove areas compared to other wetlands in the region (*SI Appendix*, Figs. S2 and S3). Mangrove-dominated coastal areas were exposed to major hurricanes (≥ Saffir–Simpson Category 3) in the study region during periods of heightened storm activity from 2004 to 2005 and 2016 to 2018 that include direct hits by Hurricanes Wilma in 2005 and Irma in 2017 (*SI Appendix*, Table S6). Impacts to mangroves that lead to decreased CO_2_ uptake include massive canopy defoliation and destruction of aboveground biomass (66–68). Prolonged flooding and hydrological disruptions can exacerbate canopy loss, prevent mangrove regeneration, and lead to landscape-level dieback and the formation of mangrove ghost forests, as evidenced by impacts shown post-Irma in 2017 (69). Recovery of NEE rates in mature mangrove forests lag up to 4 y following hurricane disturbance (43, 70). The Cape Sable region contained two hotspots of high and low net CO_2_eq rates that exemplify areas with healthy intact mangrove forest vs. low-resiliency ghost forest (Fig. 4*A*).

Though hurricanes are a major driving force, South Florida’s wetlands face several environmental and management factors influencing CO_2_eq fluxes that can be monitored via satellite-based surface reflectance. Trends in surface water, vegetative water content, and vegetation health from 2000 to 2023 derived from MODIS reflectance significantly differed among the CERP water management units (*SI Appendix*, Fig. S5). Water Conservation Areas 2A, 3A, and 3B, for example, indicate more flooding in both the wet and dry seasons than other water management units (*P* < 0.0001), and these regions also had some of the lowest overall mean wet-season trends in vegetation productivity (*P* < 0.0001). We also found that MODIS reflectance imagery captured changes occurring with the salinity across the tidal freshwater gradient (r^2^= 0.71; RMSE = 6.71 ppt), the depth of managed freshwater levels (r^2^ = 0.51; RMSE = 379.5 cm), and before and after disturbance events in areas impacted by Hurricane Irma in 2017 and by prescribed and natural fires (*SI Appendix*, *Extended Methods*).

### CO_2_ and CH_4_ Fluxes Respond to Management and Environmental Drivers.

Principal component analysis (PCA) and structural equation modeling (SEM) were used to identify the key management indicators driving trends in CO_2_ and CH_4_. PCA explores complex interactions among responses and drivers by reducing high dimensionality among closely related variables. SEM provides more explanatory power to decision makers on the direct and indirect causal pathways between observed and interdependent variables driving C fluxes (71, 72). For each of 38 AHED water management units, we summarized trends in annual CO_2_ and CH_4_ fluxes from 2000 to 2023 and mean annual fluxes from 2018 to 2023 following Hurricane Irma to test as response variables to wetland management and environmental indicators relating to water depth, disturbance, and wetland habitat (*SI Appendix*, *Extended Methods*).

**Fig. 5.**
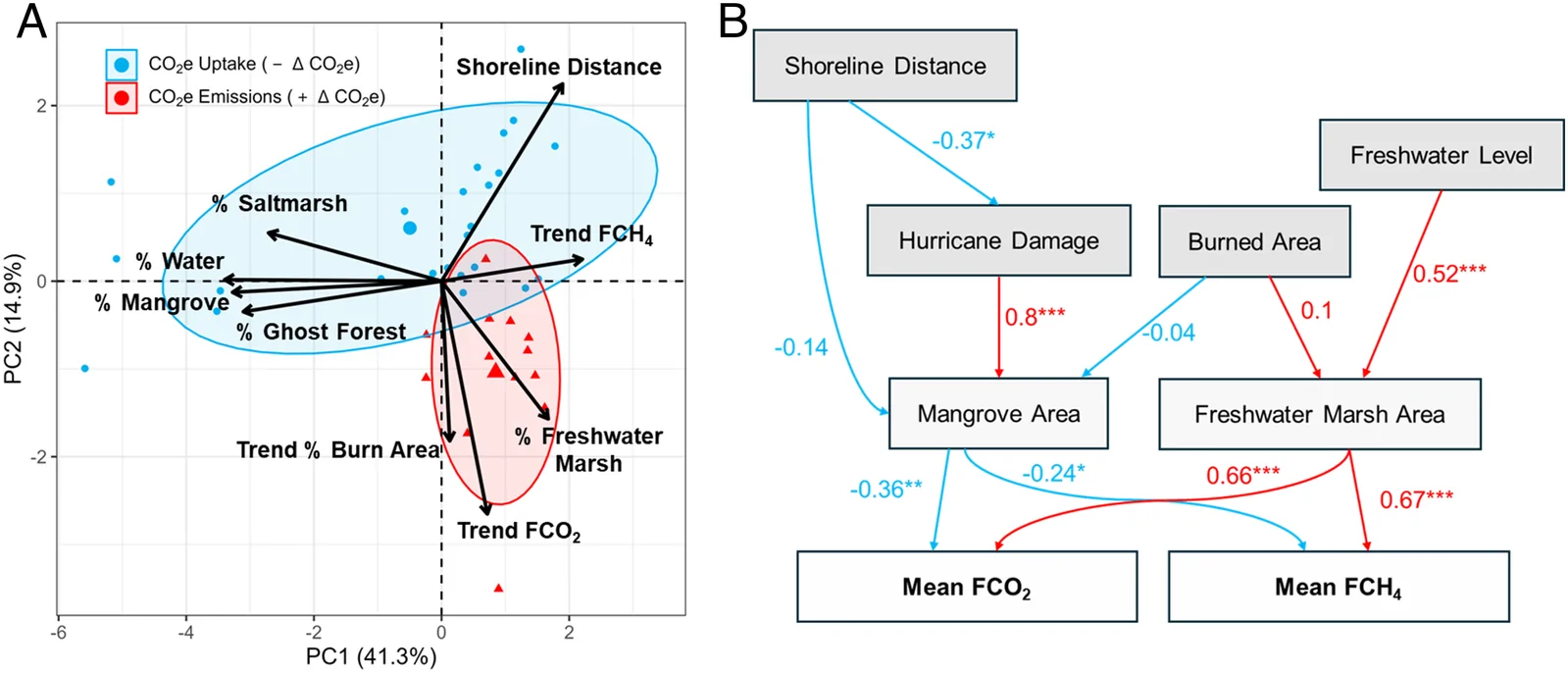
Attribution of management and environmental drivers within AHED water management units. (*A*) PC relating flux trends to wetland habitat, hurricane, and fire impacts, and a shoreline proxy for sea levels summarized for the 2000 to 2023 study period. Clusters indicate CO_2_eq uptake (blue) and emissions (red). (*B*) SEM of direct and indirect effects of dominant habitat and management factors to mean fluxes from 2019 to 2023 with the direct pathways quantified as positive (red) and negative (blue) regression coefficients with significance levels: * = 0.05, ** = 0.005, *** = 0.0001.

Clustering of the water management units showed clear dissimilarity among sites with positive and negative CO_2_eq trends (Fig. 5*A*). Principal components (PC) summarizing the indicators of management and environmental conditions together explained about 56.2% of the total variance in the dataset. In units that exhibited overall CO_2_eq emissions (+∆ CO_2_eq), positive trends in CO_2_ flux were more strongly associated with the percent area of freshwater marsh and the change in the amount of burned area over time. Freshwater marsh area directly impacts rates of mean CO_2_ flux (R^2^ = 0.66, *P* < 0.005, Fig. 5*B*). Although a direct causal pathway between burn areas and CO_2_ flux was not found, burn areas were more often associated with units containing freshwater marshes than mangroves. Freshwater marsh area was also an important factor in positive CH_4_ flux trends (Fig. 5*A*), explaining up to 67% of mean CH_4_ flux (Fig. 5*B*). Airborne fluxes also showed increased CH_4_ emissions over freshwater marshes, which were largely attributed to freshwater levels over other physical or vegetative properties (37). Conversely, intact tidal saline systems, including mangroves and saltmarshes, contributed to negative CH_4_ flux trends (Fig. 5*A*). The percent area of ghost forest and water also indicated a negative correlation with CH_4_ flux trends, but this was driven by close relationships with dominant tidal wetlands due to spatial autocorrelation. In particular, ghost forests representing hurricane damage from Irma were primarily associated with mangrove areas (R^2^ = 0.8, *P* < 0.005, Fig. 5*B*), which are more susceptible to marine stressors like hurricanes and rising sea levels based on distance to shoreline (R^2^ = −0.37, *P* = 0.009, Fig. 5*B*). Saline conditions in tidal ecosystems can reduce CH_4_ flux trends, whereas freshwater levels primarily linked to freshwater marshes likely contribute to increased plant-mediated trends in CH_4_ flux (R^2^ = 0.52, *P* < 0.005, Fig. 5*B*).

Operationalizing the complex theoretical relationships among factors driving C fluxes is an essential step toward the adaptive management of blue and teal C. The future carbon balance in these ecosystems will largely depend on how wetland vegetation changes in response to the interaction of climate change and wetland management (73). Continued SLR and salinization can lead to C release from marsh dieback, ponding, and peat collapse (74–76), or to mangrove expansion that could increase net CO_2_ capture by up to 12% (77, 78). Interacting fire and freshwater inundation can also cause habitat transitions among freshwater marsh types which could impact C uptake (79, 80).

## Conclusions

Multiscale measurements of both CO_2_ and CH_4_ fluxes are needed for a better understanding of the climate mitigation potential of blue and teal wetlands. The NASA BlueFlux campaign addresses the spatial and temporal variability in ecosystem flux using a novel combination of ground-based and airborne EC measurements of CO_2_ and CH_4_ fluxes over 23,000 km^2^ of wetlands covering the Everglades National Park (ENP), the Big Cypress National Preserve (BCNP), and surrounding areas in South Florida that represent a diversity of ecosystems managed across private, state, tribal, and federal agencies. Our upscaling approach capitalizes on flux observations from multiple scales and can incorporate future ground-based collections, regional airborne flux measurements, and advancements in satellite data to further reduce bottom–up uncertainties compared to top–down atmospheric inversion models associated with wetlands (81). Future research will also include integrating lateral, aquatic fluxes that alter the net storage of carbon in sediments (31). Satellite monitoring of carbon dynamics will be essential for addressing knowledge gaps across diverse tidal to freshwater landscapes. As all wetlands continue to face the increasing influence of human impacts related to management and climate change, such data can provide a holistic perspective on wetlands in actionable management areas to successfully reduce GHG emissions and maintain cobenefits like biodiversity. South Florida is a highly managed coastal region experiencing rapid increases in sea-level rise (82), high recurrent frequency of hurricanes causing mangrove mortality (83), and declines in mangrove extent with urbanization and fragmentation (84). This region exemplifies many other coastal regions worldwide facing the compounded effects of climate change and water management that will determine their role as natural solutions to offset global C emissions.

## Materials and Methods

### Study Area: ENP and BCNP.

The study domain focuses on ecosystems spanning a tidal freshwater gradient within the ENP, the BCNP, and surrounding South Florida region where in situ and aircraft measurements were collected as part of the BlueFlux field campaign (Fig. 1). We use contemporary distributions of the diverse wetland and upland ecosystems within ENP and BCNP that represent accurate baselines for documenting change and informing management (85, 86). For surrounding areas, we relied on the SFWMD map of Land Cover Land Use for 2017 to 2019. Our study domain contains the Everglades, Florida Bay, and the Keys regions of the CERP.

### MODIS Reflectance Data.

The MODIS collection 6.1 MCD43A4 Nadir Bidirectional Reflectance Distribution Function (BRDF)-Adjusted Reflectance (NBAR) product provides gridded, daily surface reflectance at 500-m resolution dating back to February 2000. Daily NBAR values are derived from albedo-corrected, cloud-masked MODIS Terra and Aqua morning and afternoon overpasses, with daily data representing a 16-d period of observation, weighted by quality, and corrected with a semiempirical BRDF model (87). Available NBAR reflectance bands include red (620 to 670 nm), near infrared 1 (NIR1; 841 to 876 nm), blue (459 to 479 nm), green (545 to 565 nm), NIR2 (1,230 to 1,250 nm), short-wave infrared 1 (SWIR1; 1,628 to 1,652 nm), and SWIR2 (2,105 to 2,155 nm). We accessed NBAR data through Google Earth Engine (GEE), a cloud-based platform for storing and analyzing geospatial and remotely sensed data (88). Data were filtered to the study region for February 2000 to August 2024. Additional preprocessing of the daily NBAR time series included quality masking using the MODIS collection 6.1 MCD43A2 BRDF and Albedo Quality dataset to keep pixels with good quality (bit values 0 to 3) inversion data that are not filled (89). Water masks for NBAR images and composites were created using gray-level histogram threshold selection on the NIR1 band to automatically detect the optimal value for delineating water and land (90). This allowed dynamic water masking on either daily images or monthly composites to remove inundated pixels from further analyses when appropriate. Quality and cloud filtering reduced the available daily NBAR observations by 36%. MODIS Terra Land Water Mask (MOD44W.006) data from 2000 to 2015 was used to remove open water and built-up pixels for mapping purposes (91).

We used the MODIS NBAR time series as the basis for upscaling wetland fluxes due to its global, 500-m spatial coverage of quality-controlled, BRDF-corrected daily surface reflectance since 2000. We developed models solely on surface reflectance to account for high temporal frequency and spatial resolution, to reduce prediction errors in model development, and to aid the extrapolation of flux predictions using a consistent global satellite-based time series when ancillary data is sparse (*SI Appendix*, *Extended Methods*).

### EC Tower Measures of Ecosystem Flux.

Climate-monitoring networks provide essential long-term data on carbon, water, and energy cycling in wetlands and other ecosystems. In the South Florida region, the EC tower network is operated and maintained by academic, state, and federal cooperative agreements, and composed of individual AmeriFlux principal investigators who collaborate through the Florida Coastal Everglades Long-Term Ecological Research (FCE-LTER) program (31), the United States Geological Survey (USGS) Priority Ecosystem Science Everglades Program, and the SFWMD (Fig. 1). This network monitors NEE using the eddy-covariance method to quantify vertical exchange between the land surface and the atmosphere (38, 39). At EC tower sites, aquatic, biological, and meteorological variables are sampled by the minute and gaseous fluxes at 10 or 20 Hz, and are then processed using standard methods to derive estimates of half-hourly turbulent fluxes of NEE (92).

We compiled a half-hourly time series of CO_2_ and CH_4_ fluxes from eight EC towers in South Florida that represent a range of wetland vegetation types with varying hydrology and disturbance histories (*SI Appendix*, Table S1). These towers are part of the Florida Coastal Everglades Long Term Ecological Research (FCE LTER) Program. The Shark River Slough (US-Skr; SRS6) tower sits within tidal mature riverine mangroves, including *Rhizophora mangle* (L.), *Avicennia germinans* (L.), and *Laguncularia racemosa* (L.) C.F.Gaertn, exceeding 20 m in height that are frequently impacted by hurricanes, most notably Hurricane Wilma in 2005 (41–43) and Hurricane Irma in 2017 (68). The Everglades long hydroperiod marsh (US-Elm; SRS-2) is also within the Shark River Slough, in freshwater marsh dominated by spike rush (*Eleocharis cellulosa* Torr.) and sawgrass [*Cladium mariscus subsp. jamaicense* (Crantz) Kük] (47). The Everglades short hydroperiod marsh (US-Esm; TS/Ph-1b) tower is located within the Taylor Slough in a mix of freshwater marsh habitats dominated by sawgrass and muhly grass [*Muhlenbergia capillaris* (Lam.) Trin.] (45, 46). The Taylor Slough/Panhandle (US-TaS; TS/Ph-7) tower occupies a mesohaline marsh-mangrove forest ecotone dominated by *R. mangle* scrub forest less than 2 m in height (40, 41). In the Everglades Saltwater Intrusion Marsh (US-EvM; SE-1), where saltwater intrusion and ponding in marshes are leading to mangrove encroachment, the vegetation is a changing mix of *R. mangle*, black rush (*Juncus roemerianus* Scheele), and sawgrass (44). Saltmarshes in this region include black rush, cordgrasses (*Spartina* spp.), and salt grass [*Distichlis spicata* (L.) Greene], but saltmarshes are less dominant and inland relative to mangroves in tidal habitats (93). The BCNP contains additional tower sites managed cooperatively by the U.S. Geological Survey (USGS) and the SFWMD in dominant freshwater and upland forest types of South Florida (48). These tower sites include dwarf cypress swamp (US-DCS) with stunted *Taxodium distichum* (L.) Rich. dominating sawgrass, and tall cypress swamp (US-TCS) containing mature, dense *T. distichum* forest with a mixed hardwood subcanopy (48, 49). The pine upland (US-PiU) tower site is within an open canopy slash pine (*Pinus elliottii* Engelm.) forest with scattered cypress domes, saw palmetto [*Serenoa repens* (W. Bartram) Small.], sabal palms [*Sabal palmetto* (Walter) Lodd. ex Schult. & Schult.f.], mixed hardwood understory, and groundcover containing a mix of grasses, sedges, and forbes (48, 49).

Due to differences in data sources, accessibility, data sharing policies, equipment failures, and post-processing methods, we performed additional quality control to ensure that EC tower measures of ecosystem fluxes are comparable across the entire period of data collection from different sources (*SI Appendix*, *Extended Methods*). Processed tower inputs for upscaling include daily average CO_2_ flux ± SD (μmol m^−2^ s^−1^) and daily average CH_4_ flux ± SD (nmol m^−2^ s^−1^) calculated per tower when measurements covered more than 80% of the 24-h period. Towers interspersed across the region provided long-term data of daily CO_2_ (n = 7,210) dating back to 2004 and of daily CH_4_ (n = 3,903) since 2012 (*SI Appendix*, Table S1).

### Airborne EC Measures of Ecosystem Flux.

BlueFlux deployments were conducted on 19 to 26 April 2022, 14 to 20 October 2022, 06 to 13 February 2023, 13 to 19 April 2023, and 13 to 22 July 2024 (Fig. 1). Each deployment consisted of six to eight flights, primarily during midday h 10:00 and 17:00 (LT) taken over several days. 1-Hz CARAFE measurements of CO_2_ and CH_4_ fluxes collected from a total of 30 flight days are available for georeferenced flight segments (50). We limited our analysis to CARAFE flux measurements sampled during midday (10 am to 2 pm) to match the acquisition times of MODIS. To match the 500-m resolution of MODIS, CARAFE samples within a given pixel were averaged to create gridded mean CO_2_ and CH_4_ fluxes at 500-m.

To estimate the daily integrated average CO_2_ flux from midday CARAFE samples, we capitalized on the diel tower time series. Linear relationships between midday and daily CO_2_ flux were developed from the eight EC towers used in this study (*SI Appendix*, Fig. S6), which follows the approach to scale midday CARAFE samples to 24-h average using monthly data from four EC towers coincident with flight paths (37). We convert CARAFE midday average CO_2_ flux (x; μmol m^−2^ s^−1^) to daily average CO_2_ flux (y; μmol m^−2^ s^−1^) using the updated linear fits:

[1] y=(0.27±0.002)x+(0.58±0.01).

We estimated the SE of model terms by propagating uncertainty as the SD in daily average CO_2_ flux through Eq. **1**. Daily average CH_4_ flux (nmol m^-1^ s^-1^) was estimated as the mean midday CH_4_ flux scaled by 24 h and treated as a constant throughout the day, as no significant relationship between midday-daily CH_4_ flux was detected (37). Gridded and tabular CARAFE flux inputs for upscaling include daily average CO_2_ flux ± SD (μmol m^−2^ s^−1^) and daily average CH_4_ flux ± SD (nmol m^−2^ s^−1^) linked to 500-m MODIS pixels incident with CARAFE flights. Airborne EC collected from five seasonal deployments from 2022 to 2024 yielded estimates of CO_2_ (n = 10,924) and CH_4_ (n = 10,919) fluxes at the daily, 500-m scale that transverse the study region’s ecosystems.

### Upscale Modeling.

Daily average fluxes from airborne CARAFE and EC towers were used as the basis for upscaling CO_2_ flux (μmol m^−2^ s^−1^) and CH_4_ flux (nmol m^−2^ s^−1^). Combined CARAFE and EC tower data provide improved geographic and temporal coverage of fluxes, respectively, together capturing more variability across wetland habitats, disturbances, and seasons in South Florida. Mean MODIS band reflectance (blue, green, red, NIR1, NIR2, SWIR1, and SWIR2) was sampled at 500-m for each CARAFE and EC tower flux measurement by date to serve as predictor covariates. Spectral indices were not added as covariates, as they can saturate and add no additional predictive power compared to models based solely on surface reflectance (94). Quality and cloud filtering of NBAR observations reduced flux training samples by 16%.

We developed bootstrap random forest regressions for each flux using the combined CARAFE and EC tower fluxes. Random forest was selected for its robustness to outliers and similar performance to other machine learning algorithms in predicting NEE in the ENP and BCNP (95). Random forest models were initially developed and tested on 80% of training data with cross-validation (k = 10) to tune model parameters to minimize RMS error (RMSE) using “randomForest” (v4.7-1.1; 96) and “ranger” (v0.15.1; 97) in R (98). Calibrated models were then run in a bootstrap ensemble (n = 100) approach using “Scikit-Learn” (99) in Python (100) on the NASA Center for Climate Simulation (NCCS) Discover Supercomputer to produce robust aggregated predictions of flux intensity and uncertainty. Uncertainty was estimated as the SD of the upscaling predictions in CH_4_ and CO_2_ fluxes derived from 100 bootstrap ensemble random forest models. Models were evaluated based on the variance explained (r^2^), and the RMSE and mean absolute error (MAE) of predicted fluxes fit to the measured fluxes at daily, monthly, and annual time scales. We also investigated the importance and effects of predictor covariates on individual models.

### Upscale Predictions for Landscape Net Carbon Fluxes.

We used upscale models to predict daily CO_2_ and CH_4_ as a function of MODIS NBAR reflectance, resulting in 500-m gridded daily average CO_2_ flux ± SE (μmol m^−2^ s^−1^) and daily average CH_4_ flux ± SE (nmol m^−2^ s^−1^) covering the available NBAR archive from February 2000 to August 2024. MODIS NBAR data for prediction were acquired using the NASA Application for Extracting and Exploring Analysis Ready Samples (AρρEEARS). Upscaled model flux predictions were accomplished using the NCCS Discover.

We assessed the long-term annual and seasonal trends in predicted CO_2_ and CH_4_ fluxes within the study domain, protected park areas, and AHED water management watersheds. Trend significance and magnitude were estimated using Kendall’s tau with Sen’s slope from 2000 to 2023 (59), excluding partial data for 2024. Predictions were benchmarked against towers and flight data. We estimated net annual carbon exchange for the study region according to habitat distributions in South Florida. Predictions of CO_2_ and CH_4_ fluxes were aggregated over seasonal and annual time scales within the study domain and protected areas for comparisons to GHG inventories. We used the IPCC (AR6) 100-y (GWP) value of 27.0 for nonfossil methane to convert methane emissions to CO_2_ equivalents (CO_2_eq).

### Management and Environmental Drivers.

PCA and SEM were used to identify the key management indicators driving trends in CO_2_ and CH_4_. PCA reduces high dimensionality in datasets by identifying closely related variables to explore complex interactions of response and driver variables. For each of 38 AHED water management units, we summarized trends in annual CO_2_ and CH_4_ fluxes from 2000 to 2023 as response variables to wetland management and environmental indicators in three categories: water depth, disturbance, and habitat. Distance to shore was used as a proxy of sea-level rise inundation. Water depth data across the study region were collected from the Everglades Depth Estimation Network (EDEN) and Coastal EDEN datasets (101), providing modeled and measured time series on water surface levels. We used these data to summarize trends in annual water levels (m NAVD88) for each AHED unit where data was available (n = 20). We included trends in percent burned area as a disturbance indicator, populated with annual burned area (ha) from the Monitoring Trends in Burn Severity (MTBS; 102) per AHED unit. Percent ghost forest area (ha) per unit was used as an indicator of hurricane disturbance in mangrove areas showing low recovery (*SI Appendix*, *Extended Methods*). We included the percent areas of other habitats including mangrove, salt marsh, freshwater marsh, hammock forest, human impacted, nonvegetative, other vegetative (agriculture, exotics), swamp forest, swamp scrub, swamp shrubland, swamp woodland, upland scrub, upland shrubland, upland woodland, and water, but less dominant habitats were iteratively removed when found to be nonsignificant contributors to the PC.

To explain the complex interactions of management indicators and their influence on CO_2_ and CH_4_ fluxes, we implement SEM (72). SEM is a statistical approach for describing hypothesized causal pathways that can provide more explanatory power for decision makers on the complex relationships in wetlands, such as those among abiotic conditions, disturbance, community biomass, and species richness in a coastal wetland (103). For this analysis, management indicators and fluxes were summarized as the means from 2018 to 2023 to align with available high-resolution maps of Hurricane Irma (2017) impacts for the region (67). Hurricane damage was calculated as the ratio of low-resiliency mangrove ghost forest area post-Irma to the total mangrove area pre-Irma. We designed and tested the conceptual structural models using “lavaan” (104), based on the sample covariance matrix for standardized observed input variables, which included mean CO_2_ and CH_4_ flux as endogenous variables and management indicators as exogenous variables (*SI Appendix*, Table S6 and *Extended Methods*).

## Supplementary Material

Appendix 01 (PDF)

The SI Appendix has been updated to remove W. Barclay Shoemaker from the author line.

## Data Availability

netCDF data have been deposited in ORNL DAAC (10.3334/ORNLDAAC/2404). Previously published data were used for this work (50). All other data are included in the manuscript and/or *SI Appendix*.
