# Supplementary material for: Compounded effects on wetland greenhouse gas fluxes from climate change and water management along a saline to freshwater gradient
Source: Proc Natl Acad Sci U S A. 2026 Feb 17;123(8):e2513685123. doi: 10.1073/pnas.2513685123 (PMC12933060; doi:10.1073/pnas.2513685123)
Supplement: Supplementary file 1 — Appendix 01 (PDF) [file pnas.2513685123.sapp.pdf]

PNAS

Supporting Information for

Compounded effects on wetland greenhouse gas fluxes from climate change and water management along a saline to freshwater gradient

Cheryl L. Doughty[a,b,1], Qing Ying[a,b], Eric Ward[a,b], Erin Delaria[a,c], Glenn M. Wolfe[c], Sparkle L. Malone[d], David E. Reed[d], Tiffany Troxler[e,f], John S. Kominoski[e,g], Edward Castañeda-Moya[e,2], W. Barclay Shoemaker[h], David Yannick[i], Gregory Starr[i], Steven F. Oberbauer[e,g], Abigail Barenblitt[a,b,j], Anthony Campbell[b,k], Sean Charles[l], Lola Fatoyinbo[b,3], Jonathan Gewirtzman[d], Thomas Hanisco[c], Reem Hannun[m], Stephan Kawa[c], David Lagomasino[l], Leslie Lait[c], Ayia Lindquist[b], Paul Newman[n], Peter Raymond[d], Judith Rosentreter[o], Kenneth Thornhill[p], Derrick Vaughn[d,q], and Benjamin Poulter[b,4].

***Email, corresponding author:** Cheryl Doughty, cheryl.l.doughty@nasa.gov

This PDF file includes:

Supporting text
Figures S1 to S7
Tables S1 to S8
SI References

Other supporting materials for this manuscript include the following:

Datasets S1. C. L. Doughty, et al., BlueFlux: Modeled Daily CO2 and CH4 Wetland Fluxes, Southern Florida, 2000-2024. ORNL DAAC, Oak Ridge, Tennessee, USA. https://doi.org/10.3334/ORNLDAAC/2404. Deposited 16 April 2025.
Software S1. C.L. Doughty. Blue Fluxes: tracking the balance of carbon dioxide (CO_2) uptake and methane (CH_4) emissions in South Florida's wetlands. Google Earth Engine Web App. https://ee-cdoughty.projects.earthengine.app/view/blueflux-ghg-tracker

Supporting Information Text

Extended Methods

MODIS Reflectance Data Selection

We developed models solely on MODIS NBAR surface reflectance to account for high temporal frequency and spatial resolution and reduce predicting errors in model development and to aid the extrapolation of flux predictions using a consistent global satellite-based time series when ancillary data is sparse. Satellite-based reflectance has facilitated an improved understanding of the changing extents, carbon fluxes, and net primary productivity (NPP) in wet carbon ecosystems globally (1, 2). Bottom-up approaches to extrapolate ground-based carbon metrics using satellite surface reflectance can estimate global gross primary production (GPP) with 77% accuracy (3), showing consistency with global GPP and NEE estimates from solar-induced fluorescence (SIF) and top-down atmospheric inversion approaches (4). Yet, global and regional upscaling efforts have revealed high levels of uncertainty in the tropics, especially in tropical wetlands, and in interannual fluxes and trends, which vary greatly over space and time among differing plant functional types (4–8).

EC Tower Data Processing

Half-hourly time series of CO_2 and CH_4 fluxes were collected from 8 eddy covariance towers in South Florida that represent a range of wetland vegetation types with varying hydrology and disturbance histories (SI Appendix; Table S1). Differences in data sources, accessibility, data sharing policies, equipment failures, cost, labor, and post-processing methods influence the availability of long-term EC tower data for either CO_2 and CH_4. For example, CH_4 observations were prioritized in Dwarf Cypress over Tall Cypress due to the financial and labor costs of installing a LI-7700 CH4 Analyzer (LI-COR Environmental, Lincoln NE, USA) on towers over 30 m tall. Due to these differences, we performed additional quality control to ensure that EC tower measures of ecosystem fluxes are conservative and comparable across the entire data collection period from different sources. We included data from US-PiU in our analysis because forested uplands in the Everglades are spatially autocorrelated and mixed in pixels with swamp ecosystems at the 500-m spatial scale. This data also serves as an upland constraint on CO_2 flux values that expand the boundary conditions of developed models to improve generalizability and inter-site comparisons.

We opted for half-hourly AmeriFlux BASE data products as these were the most common publicly available data provided for each tower (9). We imposed strict minimum and maximums to remove global outliers in tower datasets using the physically plausible ranges for CO_2 (-50 - 50 μmol CO_2 m^{-2} s^{-1}) and CH_4 (-500 - 4000 nmol CH_4 m^{-2} s^{-1}) following FLUXNET guidance (10). AmeriFlux quality flags (FC_SSITC_TEST, FCH4_SSITC_TEST) were used when available. We also filtered data for each site by imposing limits on momentum flux (TAU), sensible heat turbulent flux (H), standard deviation of velocity fluctuations (U_SIGMA), standard deviation of lateral velocity fluctuations (V_SIGMA), standard deviation of vertical velocity fluctuations (W_SIGMA), maximum wind speed in the averaging period (WS_MAX), friction velocity (USTAR), and latent heat turbulent flux (LE) when these were available. We did not attempt data gap-filling to avoid introducing artificial errors to measured fluxes.

Daily integrated average fluxes of CO_2 and CH_4 were calculated from the half-hourly data when tower measurements covered more than 80% of the 24-hour period and were used as the inputs for upscaling. Midday average fluxes of CO_2 and CH_4 representing mean fluxes from 10am - 2pm to align with MODIS and CARAFE acquisition times. Midday averages were regressed on

total daily averages to define linear models used in scaling CARAFE measurements to the 24-hour period (Figure S5).

Structural Equation Modeling (SEM)

Conceptual structural models were tested using “lavaan” (11), based on the sample covariance matrix for standardized observed input variables which included mean CO_2 and CH_4 flux as endogenous variables and management indicators as exogenous variables (SI Appendix, Table S7). We designed the hypothetical relationship among variables as a y-side structure representing a multivariate regression pathway model. This assumes a one-way path whereby management indicators affect carbon fluxes, not vice versa. We iteratively tested the hypothetical pathways to best describe regressions (~) and covariances (~~) from the observed data:

FCO_2 ~ Mangrove (%) + Freshwater Marsh (%) (Equation 2)
Mangrove (%) ~ Shoreline Distance (Equation 3)
Hurricane Damage ~ Shoreline Distance (Equation 4)
Mangrove (%) ~ Hurricane Damage + Burn Area (%) (Equation 5)
FCH_4 ~ Freshwater Marsh (%) + Mangrove (%) (Equation 6)
Freshwater Marsh (%) ~ Freshwater Level + Burn Area (%) (Equation 7)
FCO_2 ~~ Shoreline Distance (Equation 8)

Evaluation of MODIS reflectance as a proxy for environmental drivers

Water Cover, Depth, and Management

To assess long-term trends in surface water we created mean monthly image composites of the normalized difference water index (NDWI; 12) from the filtered daily NBAR data. Trends in NDWI were calculated separately for the wet season (May - October) and dry season (November - April). The magnitude and significance of long-term trends were calculated using Sen’s Slope on a per pixel basis to assess seasonal changes in surface water from year to year. Trends were similarly estimated for NDVI, except that monthly water masks were applied first to remove the effects of water to assess long-term trends in vegetation greenness. We tested for differences in NDWI and NDVI trends among the 38 AHED water management units spanning the study area, which contains large preserved wetland areas. Significant differences in the mean trends for each region were assessed using a Kruskal-Wallis rank sum test.

As part of CERP efforts, water levels are highly managed and monitored. The Everglades Depth Estimation Network (EDEN) is an integrated network of real-time water level monitoring and elevation-based water-surface modeling for freshwater wetlands in South Florida (13–15). EDEN V2 provides daily gridded data at 400 m on predicted water surfaces from 2000 to present with reported overall root mean square error (RMSE) of 4.6 cm NAVD88 (15, 16). We acquired daily EDEN water depths from 2000 - 2023 and resampled data using bilinear interpolation to match the 500-m scale of MODIS NBAR. We then compared the daily mean EDEN water depths to the daily NBAR surface reflectance on a per-pixel basis. Random forest models were used to test how well NBAR reflectance explained variations in water depths predicted by EDEN. Random forest is a common machine learning algorithm used for classification and regression in remote sensing applications (17), able to enhance prediction accuracy and reduce overfitting through an approach that ensembles multiple decision trees from random subsets of training data (18). For management units beyond the EDEN grid, Coastal EDEN sites provide additional water level data available through USGS water level gauges. Daily mean, min, and max gauge height of tidal water levels in ft NAVD88 were acquired through USGS Water Resources.

Salinity

To test whether NBAR captures the effect of salinity in intertidal wetlands, we used in situ porewater and surface salinity measurements collected by the FCE LTER from 2000 - 2023 (SI Appendix, Table S8). We calculated mean daily salinity for each unique measurement location to compare to corresponding daily NBAR reflectance values. We used a random forest regression model to test if NBAR reflectance can explain variations in salinity.

Hurricane and Fire Disturbance

Hurricanes and fires are common pulse disturbances that have significant impacts on ecosystems in South Florida. This region experiences a high recurrence frequency of hurricanes (19). Impacts caused by the wind damage and storm surge associated with Hurricane Irma in September 2017, for example, illustrates varying levels of mangrove resilience among fringe and interior mangroves with hotspots (pixel clusters) of mangrove loss occurring in areas with prolonged flooding (20). These areas with the highest disturbance from Hurricane Irma have seen large diebacks in mangroves with little regrowth (21). We selected low, intermediate, and high resilience areas from Lagomasino et al. 2021 to represent a gradient of disturbance ranging from "ghost" forest (high disturbance) to intact forest (low disturbance). Levels of hurricane disturbance were assessed per resilience class using a t-test of pre- and post-Irma reflectance values extracted from MODIS NBAR.

Fire occurs naturally and is a management practice used in South Florida to maintain the vegetative mosaic of the Everglades, coastal prairie, and upland forests of a dynamic ecosystem (22, 23). Active fires, burn scars, and fire histories across the landscape are identifiable using satellite surface reflectance (24, 25). We used the fires delineated in the Monitoring Trends in Burn Severity (MTBS; 26) dataset to extract pre- and post-fire NBAR reflectance for over 800 prescribed and managed burn areas based on ignition dates since 2000. Differences pre- and post-fire were assessed using a paired t-test on band reflectance. The amount of annual burned areas within each AHED water management unit were also estimated from the MTBS data.

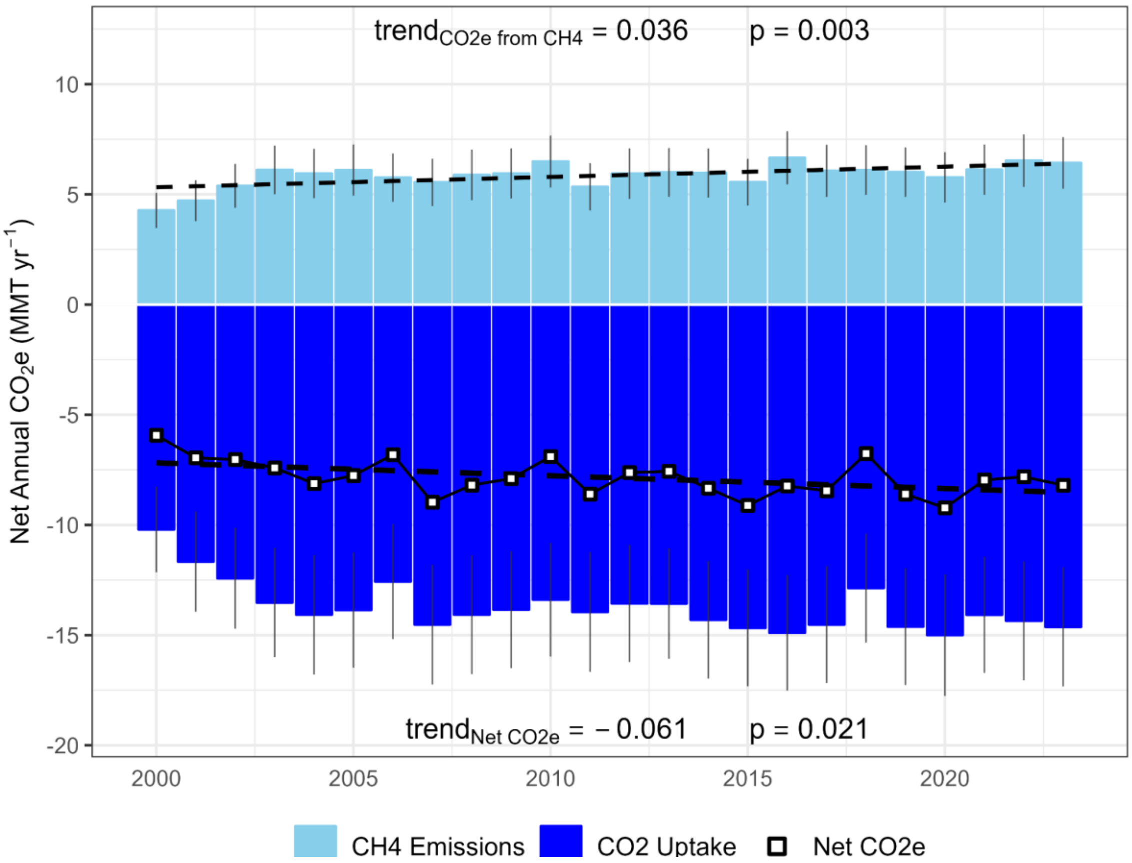

Fig. S1. Regional timeseries and trends in CO_2eq from CO_2 and CH_4 fluxes from 2000 to 2023.

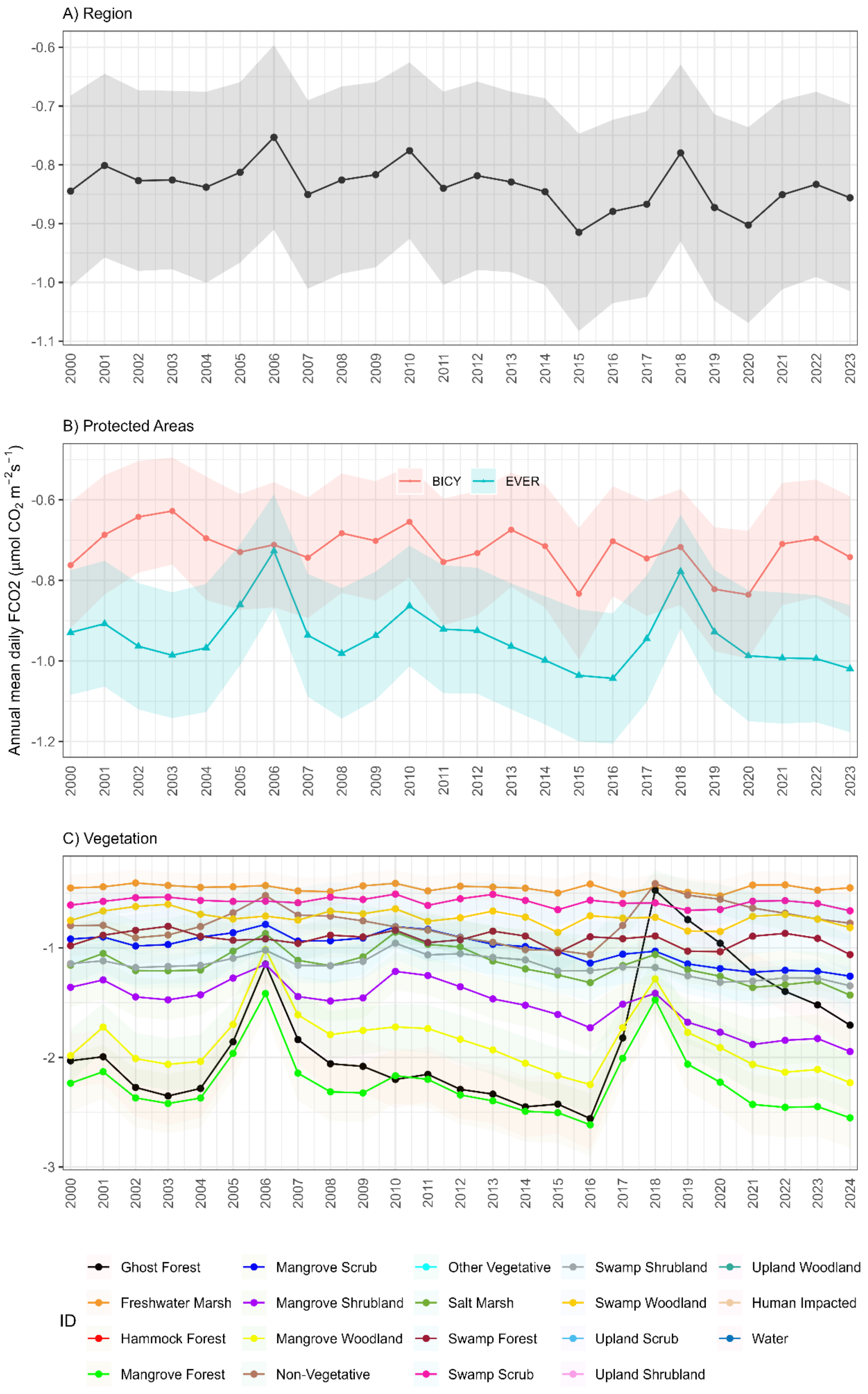

Fig. S2. Predicted mean annual CO_2 flux time series estimated for **A** the study domain, **B** Big Cypress National Preserve and Everglades National Park protected areas, and **C** land cover type.

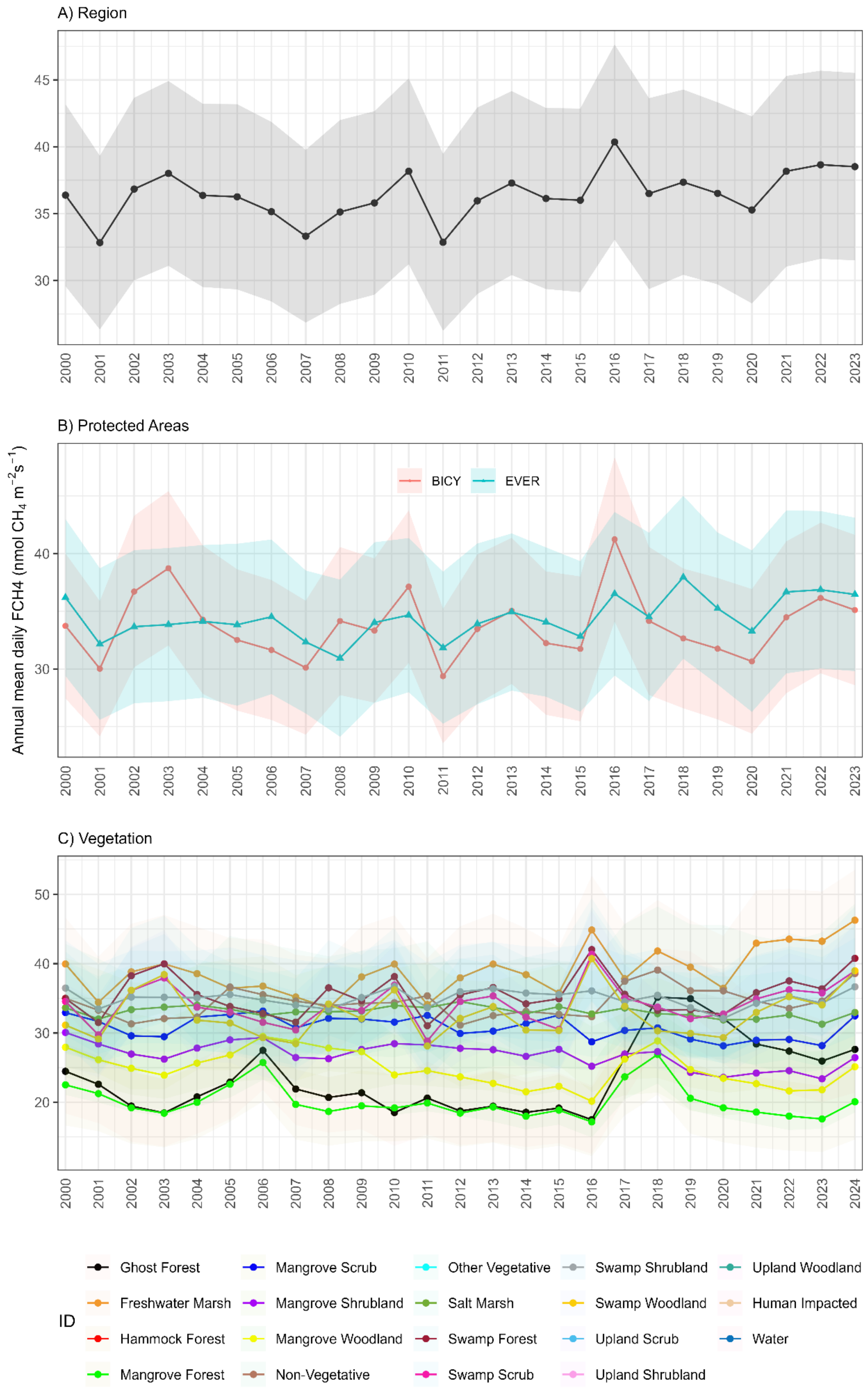

Fig. S3. Predicted mean annual CH_4 flux time series estimated for **A** the study domain, **B** Big Cypress National Preserve and Everglades National Park protected areas, and **C** land cover type.

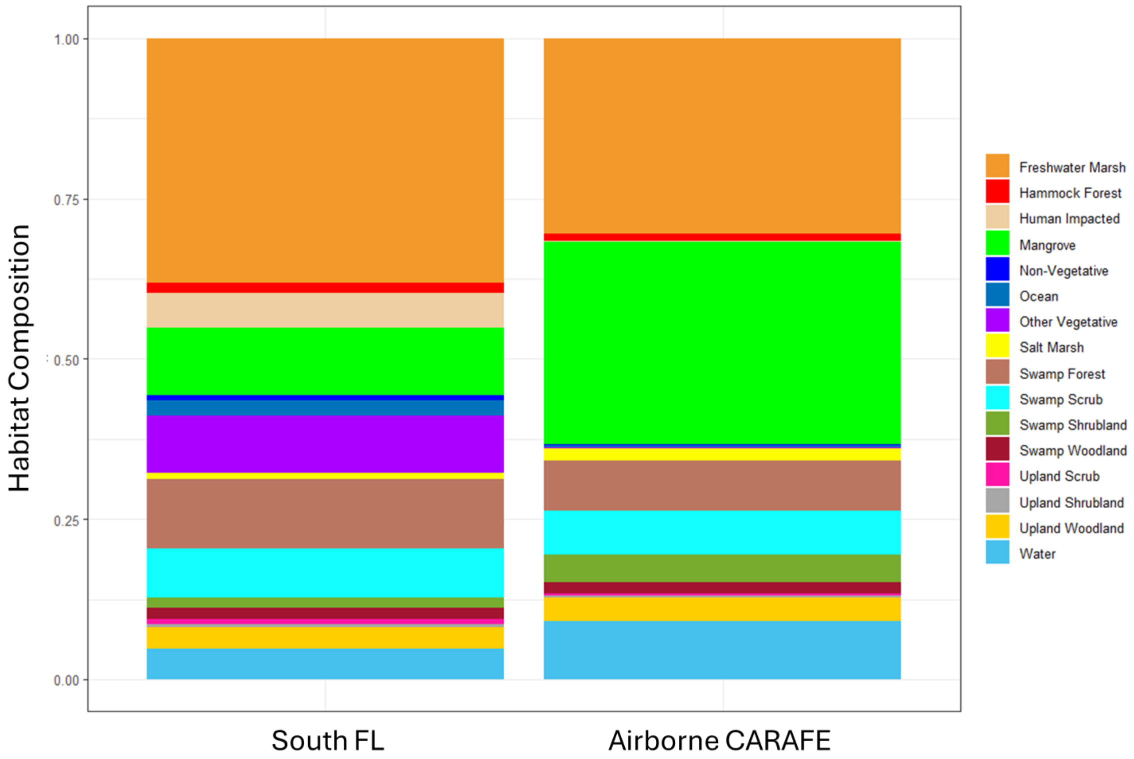

Fig. S4. Habitat composition of ecosystems measured for the South Florida study domain and the areas sampled by airborne CARAFE.

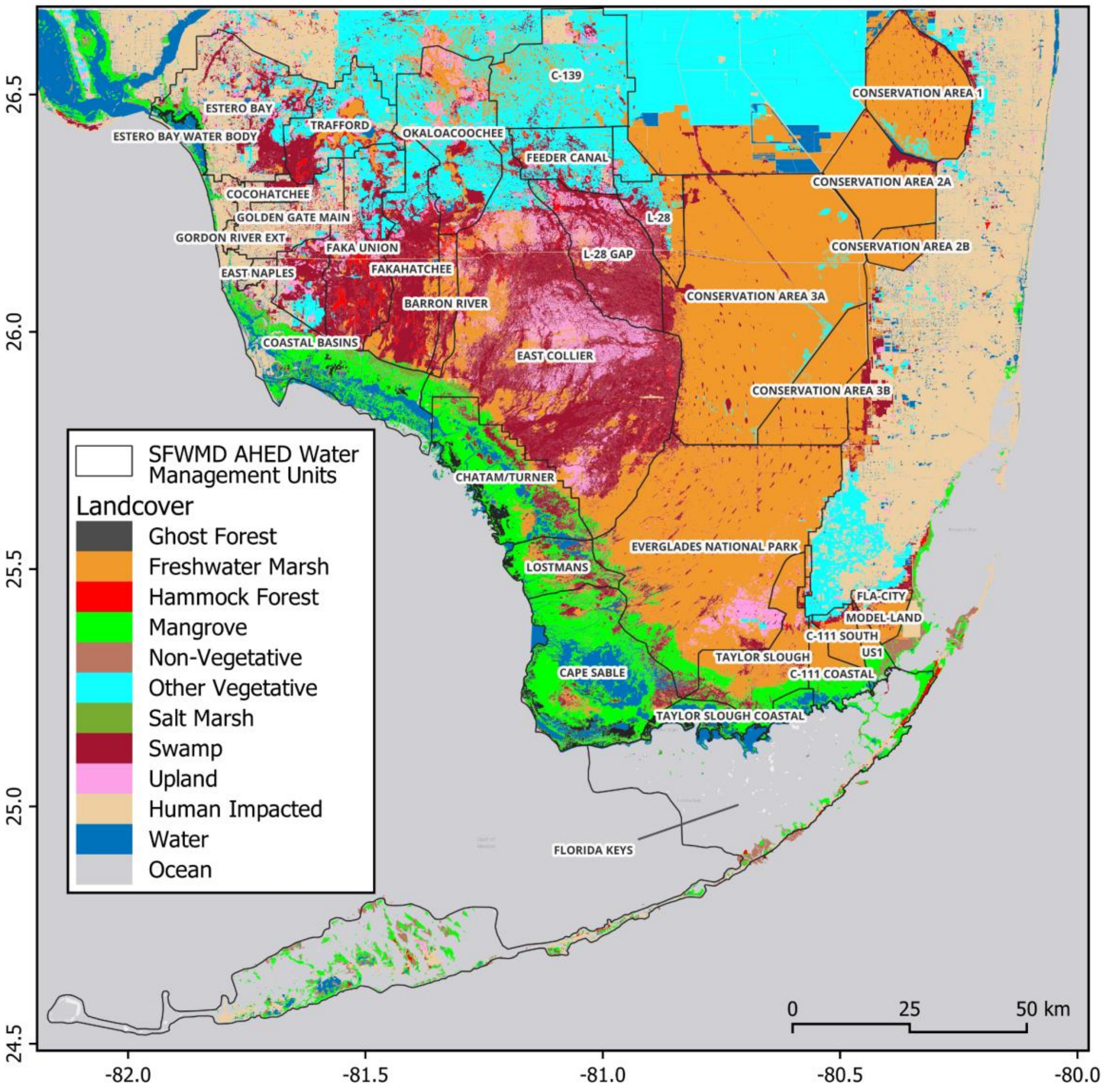

Fig. S5. The BlueFlux study domain covers the South Florida Water Management District's (SFWMD) Arc Hydro Enhanced Database (AHED) on water management units in the Comprehensive Everglades Restoration Plan (CERP). Tidal to freshwater wetland distributions were adapted from the Everglades National Park (Ruiz et al. 2021a, 2021b), the Big Cypress National Preserve (Whelan et al. 2020a, 2020b), and in surrounding wetland ecosystems (SFWMD 2018). Ghost forest distributions represent mangroves most impacted by Hurricane Irma (Lagomasino et al. 2021).

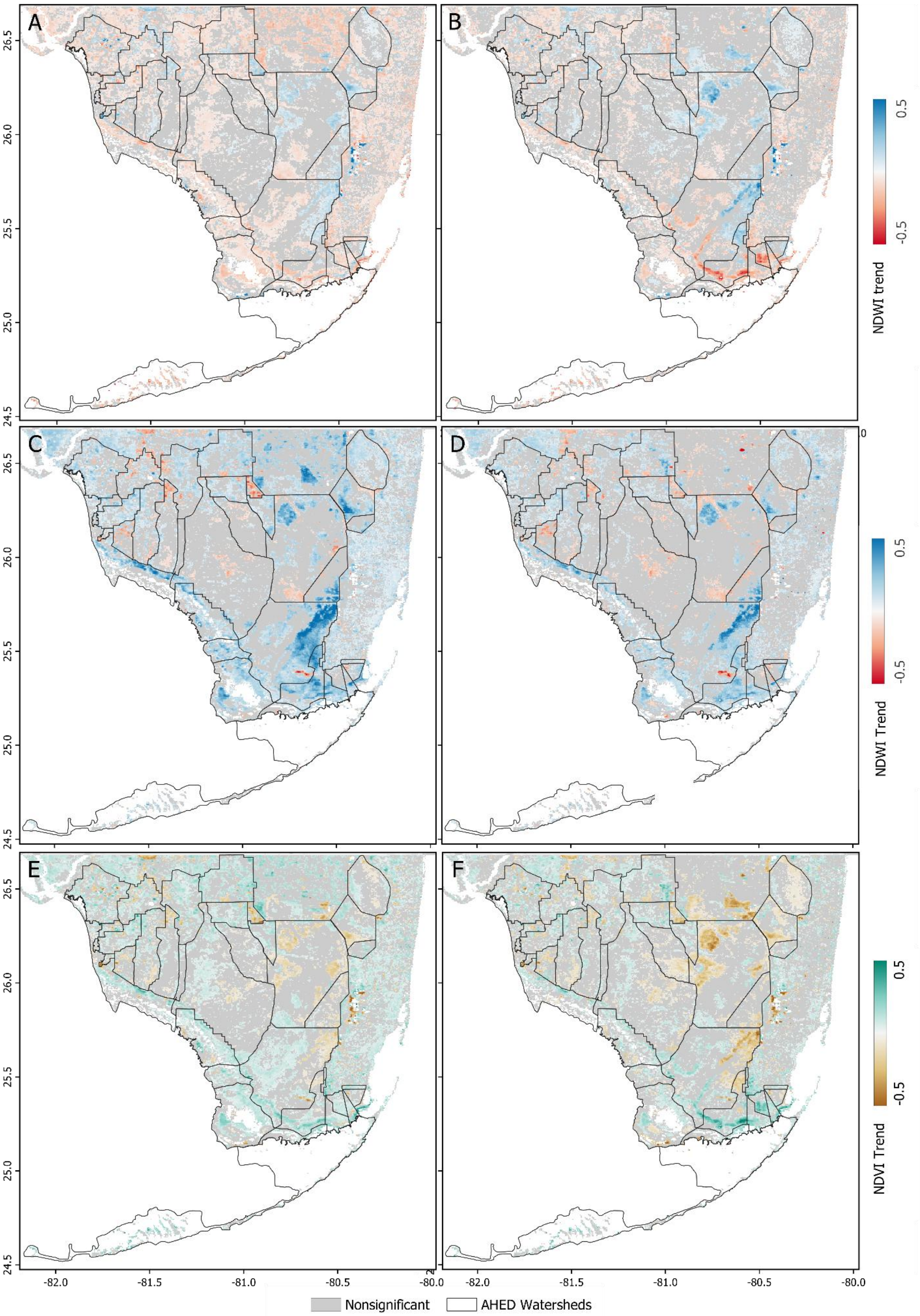

Fig. S6. Significant trends in **A** dry-season (November - April) and **B** wet-season (May-October) surface water content ($NDWI_{McFeeters}$), **C** dry-season and **D** wet-season vegetation water content ($NDWI_{Gao}$), and **E** dry-season and **F** wet-season vegetation greenness (NDVI).

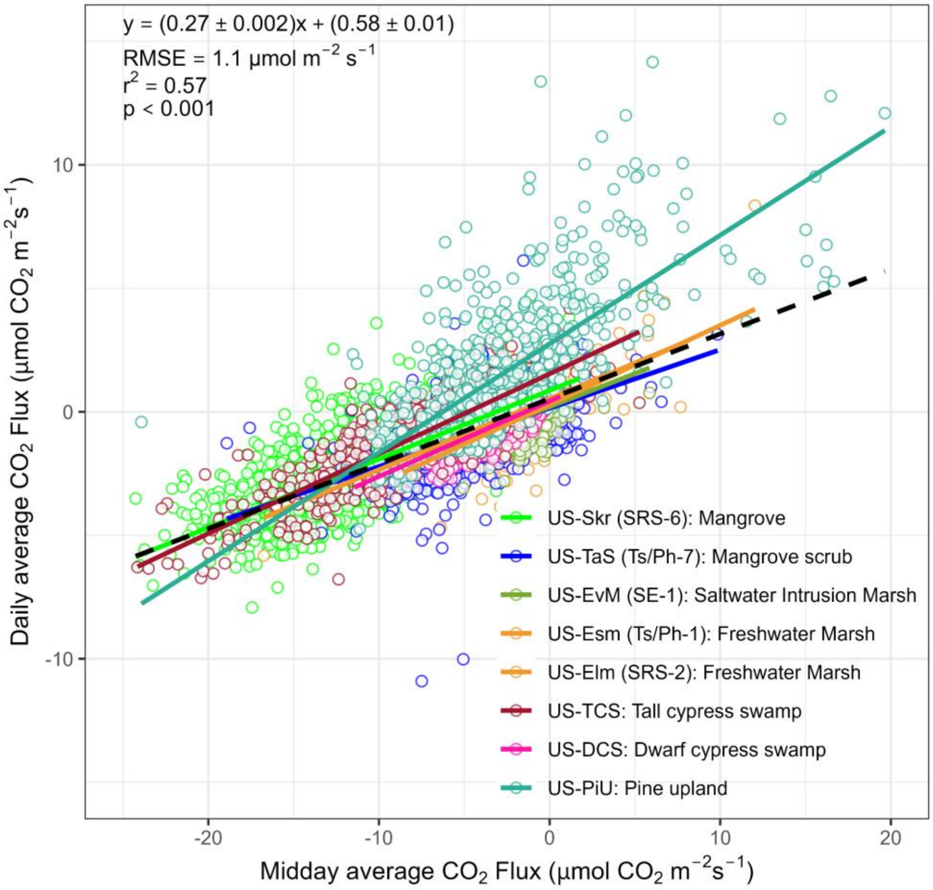

Fig. S7. Linear relationship of estimated midday (10a-2p) CO_2 flux to total daily CO_2 flux for eight EC towers in South Florida.

Table S1. Eddy covariance (EC) tower site descriptions and data availability for half-hourly CO_2 and CH_4 fluxes from AmeriFlux (https://ameriflux.lbl.gov), FCE-LTER (https://fcelter.fiu.edu), and USGS GEPES.

Tower ID	Ameriflux Tower Name (Lat, Lon) Elevation	Habitat and Vegetation Description	Hydrography	Data Period	Fluxes Measured	Years Available	Data Source	Data Citation
US-Skr (SRS-6)	Shark River Slough Tower SRS-6 Everglades (25.3629, -81.0776) 0 m	Riverine mangrove forest; Mangrove wetland, large stature	Seasonally driven freshwater inputs and tidally driven oceanic inputs	1	CO_2	2004 - 2011	AmeriFlux BASE/ Legacy	Fuentes (27)<br>Malone et al. (28)<br>Malone et al. (29)
				2	CO_2<br>CH_4	2017 - 2023	FCE-LTER	Provisional data provided by tower PI Sparkle Malone<br>Ameriflux citation coming soon*
US-Elm (SRS-2)	Everglades long hydroperiod marsh (25.5519, -80.7826) 0.77 m	Freshwater marsh; Sawgrass dominated marsh interspersed with Eleocharis/Panicum slough	Seasonally driven freshwater sheetflow	1	CO_2	2008 - 2014	AmeriFlux BASE	Starr and Oberbauer (30)<br>Malone et al. (28)<br>Malone et al. (29)
				2	CO_2<br>CH_4	2016 - 2023	FCE-LTER	Provisional data provided by tower PI Starr, Oberbauer, Yannick
US-Esm (TS/Ph-1)	Everglades short hydroperiod marsh (25.4379, -80.5946) 1.07 m	Freshwater marsh; Sparse sawgrass marsh	Seasonally driven freshwater sheetflow	1	CO_2	2008 - 2015	AmeriFlux BASE	Starr and Oberbauer (30)<br>Malone et al. (28)<br>Malone et al. (29)
				2	CO_2<br>CH_4	2016 - 2023	FCE-LTER	Provisional data provided by tower PI Starr, Oberbauer,

Tower ID	Ameriflux Tower Name (Lat, Lon) Elevation	Habitat and Vegetation Description	Hydrography	Data Period	Fluxes Measured	Years Available	Data Source	Data Citation
								Yannick
US-TaS (TS/Ph-7)	Taylor Slough/ Panhandle (25.1908, -80.6391) 3 m	Mangrove scrub forest; Mangrove wetland, low/dwarf stature	Mesohaline; Seasonally driven freshwater inputs and wind-driven estuarine inputs	1	CO_2	2016 - 2023	AmeriFlux BASE	Malone and Troxler (31)<br>Malone et al. (29)
US-EvM (SE-1)	Saltwater Intrusion Marsh South eastern Everglades (25.3539, -80.3810) 0.33 m	Saltwater intrusion marsh; Sawgrass transitioning to mangrove wetland, low/dwarf stature	Oligohaline ecotone transitioning due to saltwater intrusion and sea level rise	1	CO_2<br>CH_4	2020 - 2023	AmeriFlux BASE	Starr and Oberbuer (32)<br>Malone et al. (28)<br>Malone et al. (29)<br>Provisional data provided by tower PI Starr, Oberbauer, Yannick
US-DCS	Dwarf Cypress Swamp (25.7621, -80.8934) 2.05 m	Dwarf cypress swamp, low/dwarf stature with sawgrass	Seasonally driven freshwater sheetflow	1	CO_2, CH_4, H	2012 - 2018	AmeriFlux	Provisional data provided by tower PI Shoemaker<br>USGS Priority Ecosystem Science Everglades Program<br>Shoemaker et al. (33)<br>Shoemaker et al. (34)
US-TCS	Tall Cypress Swamp	mature, cypress forest	Seasonally driven	1	CO_2, H	2012 - 2018	AmeriFlux	Provisional data provided by tower PI

Tower ID	Ameriflux Tower Name (Lat, Lon) Elevation	Habitat and Vegetation Description	Hydrography	Data Period	Fluxes Measured	Years Available	Data Source	Data Citation
	(25.8221, -81.1017) 1.2 m	with mixed hardwood subcanopy	freshwater sheetflow					Shoemaker Shoemaker et al. (33) Shoemaker et al. (34)
US-PiU	Pine Upland (26.0004, -80.9261) 3.04	mixed lowland pine, extensive open-canopy pine forest with mixed understory	Seasonally driven precipitation and freshwater sheetflow	1	CO_2, H	2012 - 2018	AmeriFlux	Provisional data provided by tower PI Shoemaker Shoemaker et al. (33) Shoemaker et al. (34)

Table S2. Random Forest Out-of-bag (OOB) error comparison from initial model development based on flux training data subsets of multisource airborne and tower eddy covariance (EC) observations.

	**Model**	**r^2**	**RMSE**	**MAE**
Daily mean CO_2 flux ($\mu mol\ m^{-2}\ s^{-1}$)	CARAFE (n=10,924)	0.29	1.20	0.81
	TOWERS (n=7,210)	0.66	1.55	0.91
	CARAFE + TOWERS (n=18,134)	0.48	1.42	0.89
Daily mean CH_4 flux ($nmol\ m^{-2}\ s^{-1}$)	CARAFE (n=10,919)	0.23	48.1	30.9
	TOWERS (n=3,903)	0.41	35.5	22.0
	CARAFE + TOWERS (n=14,822)	0.25	46.2	29.4

Table S3. Site-based linear models for flux validation from ground and airborne observations for mean annual CH_4 flux (nmol m^{-2} s^{-1} ± SD), and mean annual CO_2 flux (μmol m^{-2} s^{-1}).

siteDesc	flux	R^2	p-value	RMSE
US-Skr (SRS-6): Mangrove	CH_4	0.32	0.317	4.66
US-EvM (SE-1): Saltwater Intrusion Marsh	CH_4	0.05	0.64	2.4
US-Esm (TS/Ph-1): Freshwater Marsh	CH_4	0.43	0.11	3.63
US-Elm (SRS-2): Freshwater Marsh	CH_4	0.25	0.21	2.05
US-DCS: Dwarf cypress swamp	CH_4	0.89	0.005	2.79
US-Skr (SRS-6): Mangrove	CO_2	0.49	0.008	0.47
US-TaS (TS/Ph-7): Mangrove scrub	CO_2	0.01	0.858	0.14
US-EvM (SE-1): Saltwater Intrusion Marsh	CO_2	0.85	0.003	0.02
US-Esm (TS/Ph-1): Freshwater Marsh	CO_2	0.14	0.208	0.08
US-Elm (SRS-2): Freshwater Marsh	CO_2	0.04	0.506	0.1
US-TCS: Tall cypress swamp	CO_2	0.34	0.223	0.32
US-DCS: Dwarf cypress swamp	CO_2	0.97	0.0003	0.03
US-PiU: Pine upland	CO_2	0.27	0.365	0.07

Table S4. Mean annual Emission Factors (EFs) for carbon dioxide and methane estimated where wetland vegetation has been consistent from 2000 - 2023. Annual methane to carbon dioxide offsets are calculated as $FCH_4:FCO_2$ ratios in units of grams of carbon (g C m^{-2} y^{-1}) and CO_2eq (MMT CO_2eq yr^{-1}). Dominant vegetation change classes at the 500-m scale were obtained from the South Florida Water Management District (SFWMD) maps of Land Cover Land Use for 1999 and 2017-2019.

**Classes**	**mol m^{-2} s^{-1}**		**g C m^{-2} y^{-1}**			**MMT CO_2eq yr^{-1}**		
	**FCO_2 (µmol m^{-2} s^{-1})**	**FCH_4 (nmol m^{-2} s^{-1})**	**FCO_2**	**FCH_4**	**Offset Ratio**	**CO_2eq from FCO_2**	**CO_2eq from FCH_4**	**Offset Ratio**
Stable Freshwater Marsh	-0.49 ± 0.13	40.52 ± 7.04	-185.60 ± 49.24	15.35 ± 2.67	0.083	-4.15 ± 1.1	3.4 ± 0.59	0.82
Stable Mangrove	-1.57 ± 0.20	26.43 ± 6.01	-594.68 ± 75.76	10.01 ± 2.28	0.017	-3.57 ± 0.46	0.58 ± 0.13	0.16
Stable Saltmarsh	-1.06 ± 0.16	33.24 ± 6.61	-401.51 ± 60.60	12.59 ± 2.50	0.031	-0.37 ± 0.06	0.11 ± 0.02	0.30
Stable Swamp	-1.05 ± 0.18	34.83 ± 6.90	-397.72 ± 68.18	13.19 ± 2.61	0.033	-4 ± 0.68	1.31 ± 0.26	0.33

Table S5. Modeled emission factors (EF) for CO_2 and CH_4 from select wetland vegetation. Classes represent higher thematic levels available in South Florida Water Management District (SFWMD) map of Land Cover Land Use for 1999 to 2017-2019.

Select Vegetation	Mean Annual CO_2eq from CH_4 (MMT yr^{-1})	Mean Annual CO_2eq from CO_2 (MMT yr^{-1})
Freshwater Marshes-Sawgrass	1.7 ± 0.1	-1.57 ± 0.13
Freshwater Marshes / Graminoid Prairie	0.8 ± 0.1	-0.73 ± 0.07
Mixed Shrubs	0.1 ± 0.01	-0.22 ± 0.01
Cypress	0.01 ± 0.002	-0.02 ± 0.002

Table S6. NOAA Historical Hurricane Tracks for Major Storm Events in South Florida 2000 - 2023 with intense periods of major hurricanes highlighted.

Storm Name	Start Date	End Date	Maximum Wind Speed	Minimum Pressure (MB)	Max Category
IDALIA 2023	2023-8-26	2023-9-8	115	942	H4
NICOLE 2022	2022-11-6	2022-11-11	65	980	H1
IAN 2022	2022-9-22	2022-10-1	140	937	H5
ELSA 2021	2021-6-30	2021-7-10	75	991	H1
ETA 2020	2020-10-31	2020-11-14	130	922	H4
SALLY 2020	2020-9-11	2020-9-18	95	965	H2
MICHAEL 2018	2018-10-6	2018-10-15	140	919	H5
IRMA 2017	2017-8-30	2017-9-13	155	914	H5
MATTHEW 2016	2016-9-28	2016-10-10	145	934	H5
HERMINE 2016	2016-8-28	2016-9-8	70	981	H1
WILMA 2005	2005-10-15	2005-10-26	160	882	H5
RITA 2005	2005-9-18	2005-9-26	155	895	H5
KATRINA 2005	2005-8-23	2005-8-31	150	902	H5
DENNIS 2005	2005-7-4	2005-7-18	130	930	H4
JEANNE 2004	2004-9-13	2004-9-29	105	950	H3
IVAN 2004	2004-9-2	2004-9-24	145	910	H5
FRANCES 2004	2004-8-25	2004-9-10	125	935	H4
CHARLEY 2004	2004-8-9	2004-8-15	130	941	H4
GORDON 2000	2000-9-14	2000-9-21	70	981	H1

Table S7. SEM variables and definitions measured for each AHED water management unit

**Indicator Category**	**Variable Type**	**Management Indicators (Latent, unmeasured variables)**	**Observed (Measured) Variables**
Response	Endogenous (dependent)	CH_4 Flux	Mean annual CH_4 Flux (nmol m-2 s-1)
Response	Endogenous (dependent)	CO_2 Flux	Mean annual CO_2 Flux (umol m-2 s-1)
Management	Exogenous (independent)	Freshwater Level	Mean water level (m)
Management	Exogenous (independent)	Fire disturbance	Total natural and prescribed fire area (%)
Environment	Exogenous (independent)	Salinity, tidal inundation, SLR vulnerability	Mean Distance to Shoreline (m)
Environment	Exogenous (independent)	Hurricane disturbance	Hurricane Damage Ratio (%)
Environment	Exogenous (independent)	Wetland vegetation	Mangrove and freshwater marsh Habitat Area (%)

Table S8. FCE LTER Salinity Datasets

Name	Period	Measured	Citation
Abiotic monitoring of physical characteristics in porewaters and surface waters of mangrove forests from the Shark River Slough and Taylor Slough, Everglades National Park (FCE LTER), South Florida, USA, December 2000 - ongoing	2000-12-15 to 2023-10-29	Porewater Salinity	Castañeda-Moya, E., J. Kominoski, V. Rivera-Monroy, C. Reisa. 2025. Abiotic monitoring of physical characteristics in porewaters and surface waters of mangrove forests from the Shark River Slough and Taylor Slough, Everglades National Park (FCE LTER), South Florida, USA, December 2000 - ongoing. Environmental Data Initiative. https://doi.org/10.6073/pasta/657c7189d38656e178b1c08bfa404b2a.
Water Quality Data (Porewater) from the Shark River Slough, Everglades National Park (FCE LTER), Florida, USA, January 2001 - ongoing	2001-01-01 to 2022-11-01	Porewater Salinity	Gaiser, E., D. Childers, R. Travieso. 2023. Water Quality Data (Porewater) from the Shark River Slough, Everglades National Park (FCE LTER), Florida, USA, January 2001 - ongoing. Environmental Data Initiative. https://doi.org/10.6073/pasta/5ac956aa74367024e592c201e5f72721.
Water Quality Data (Rainfall-driven autosampler) from the Shark River Slough, Everglades National Park (FCE LTER), Florida, USA, June 2003 - ongoing	2003-03-27 to 2022-12-16	Surface Salinity	Kominoski, J., E. Gaiser, D. Childers, R. Travieso. 2023. Water Quality Data (Rainfall-driven autosampler) from the Shark River Slough, Everglades National Park (FCE LTER), Florida, USA, June 2003 - ongoing. Environmental Data Initiative. https://doi.org/10.6073/pasta/354993e29b6156a47ea629cf75931153.
Water Quality Data (Extensive) from the Shark River Slough, Everglades National Park (FCE LTER), Florida, USA, October 2000 - ongoing	2000-10-31 to 2022-12-30	Surface Salinity	Gaiser, E., D. Childers, R. Travieso. 2023. Water Quality Data (Extensive) from the Shark River Slough, Everglades National Park (FCE LTER), Florida, USA, October 2000 - ongoing. Environmental Data Initiative. https://doi.org/10.6073/pasta/e2c4fde5c0568398c9d8d107bcf907ec.
Water Quality Data (Grab Samples) from the Shark River Slough, Everglades National Park (FCE LTER), Florida, USA, May 2001 - ongoing	2001-05-23 to 2022-12-06	Surface Salinity	Gaiser, E., D. Childers, R. Travieso. 2023. Water Quality Data (Grab Samples) from the Shark River Slough, Everglades National Park (FCE LTER), Florida, USA, May 2001 - ongoing. Environmental Data Initiative. https://doi.org/10.6073/pasta/29ded9394cc8196632d23aaee58e0422.
Water Quality Data (Extensive) from the Taylor Slough, Everglades National Park (FCE LTER), Florida, USA, July 1999 - ongoing	1999-07-29 to 2022-12-31	Surface Salinity	Troxler, T. 2023. Water Quality Data (Extensive) from the Taylor Slough, Everglades National Park (FCE LTER), Florida, USA, July 1999 - ongoing. Environmental Data Initiative. https://doi.org/10.6073/pasta/1d53e1d8535de6789e9ba53b14926297.
Water Quality Data (Grab Samples) from the Taylor Slough, Everglades National Park (FCE), Florida, USA, September 1999 - ongoing	1999-09-13 to 2022-12-07	Surface Salinity	Troxler, T. 2023. Water Quality Data (Grab Samples) from the Taylor Slough, Everglades National Park (FCE), Florida, USA, September 1999 - ongoing. Environmental Data Initiative. https://doi.org/10.6073/pasta/9c2d7b049da2ab0bdcd3831e610861d1.

Water Quality Data (Extensive) from the Taylor Slough, Everglades National Park (FCE LTER), Florida, USA, April 1996 - ongoing	1996-04-07 to 2022-12-31	Surface Salinity	Troxler, T. 2023. Water Quality Data (Extensive) from the Taylor Slough, Everglades National Park (FCE LTER), Florida, USA, April 1996 - ongoing. Environmental Data Initiative. https://doi.org/10.6073/pasta/a4fb94408a8798c99b5c1449c7559582.
Water Quality Data (Grab Samples) from the Taylor Slough, Everglades National Park (FCE LTER), Florida, USA, May 2001 - ongoing	2001-05-30 to 2022-12-21	Surface Salinity	Troxler, T., D. Childers. 2023. Water Quality Data (Grab Samples) from the Taylor Slough, Everglades National Park (FCE LTER), Florida, USA, May 2001 - ongoing. Environmental Data Initiative. https://doi.org/10.6073/pasta/9ce4c3b60f48e7e40d19f7b86d7d384a.
Water Quality Data (Porewater) from the Taylor Slough, just outside Everglades National Park (FCE), from August 1998 to October 2006	1998-08-01 to 2006-10-01	Porewater Salinity	Troxler, T., D. Childers. 2008. Water Quality Data (Porewater) from the Taylor Slough, just outside Everglades National Park (FCE), from August 1998 to October 2006. Environmental Data Initiative. https://doi.org/10.6073/pasta/1c4f9019e3dc4306b17a067f455430ad.
Water Quality Data (Grab Samples) from the Taylor Slough, just outside Everglades National Park (FCE), for August 1998 to November 2006	1998-08-11 to 2006-11-15	Surface Salinity	Troxler, T., D. Childers. 2008. Water Quality Data (Grab Samples) from the Taylor Slough, just outside Everglades National Park (FCE), for August 1998 to November 2006. Environmental Data Initiative. https://doi.org/10.6073/pasta/cd96927a753e84af3d9d2a07b02fa322.
Water Quality Data (Extensive) from the Taylor Slough, just outside Everglades National Park (FCE), from August 1998 to December 2006	1998-08-19 to 2006-12-03	Surface Salinity	Troxler, T., D. Childers. 2019. Water Quality Data (Extensive) from the Taylor Slough, just outside Everglades National Park (FCE), from August 1998 to December 2006. Environmental Data Initiative. https://doi.org/10.6073/pasta/986977091d9ff18aac52ea1c4886e64b.
Surface Water Quality Monitoring Data collected in South Florida Coastal Waters (FCE LTER), Florida, USA, June 1989-ongoing	1989-06-27 to 2022-12-08	Surface Salinity	Briceno, H. 2023. Surface Water Quality Monitoring Data collected in South Florida Coastal Waters (FCE LTER), Florida, USA, June 1989-ongoing. Environmental Data Initiative. https://doi.org/10.6073/pasta/f45fbf88dcf1f78f0d74c1dbdaaa8c7d.
Florida Bay Physical Data, Everglades National Park (FCE LTER), Florida, USA, September 2000 - ongoing	2000-09-10 to 2023-06-05	Surface Salinity	Fourqurean, J. 2023. Florida Bay Physical Data, Everglades National Park (FCE LTER), Florida, USA, September 2000 - ongoing. Environmental Data Initiative. https://doi.org/10.6073/pasta/3b08e9c120d28e705e3c90db0a21875f.

SI References

1. S. W. J. Canty, *et al.*, Implications of improved remote sensing capabilities on blue carbon quantification. *Estuarine, Coastal and Shelf Science* **319**, 109275 (2025).
2. A. D. Campbell, *et al.*, A review of carbon monitoring in wet carbon systems using remote sensing. *Environ. Res. Lett.* **17**, 025009 (2022).
3. J. Joiner, *et al.*, Estimation of Terrestrial Global Gross Primary Production (GPP) with Satellite Data-Driven Models and Eddy Covariance Flux Data. *Remote Sensing* **10**, 1346 (2018).
4. M. Jung, *et al.*, Scaling carbon fluxes from eddy covariance sites to globe: synthesis and evaluation of the FLUXCOM approach. *Biogeosciences* **17**, 1343–1365 (2020).
5. G. McNicol, *et al.*, Upscaling Wetland Methane Emissions From the FLUXNET-CH4 Eddy Covariance Network (UpCH4 v1.0): Model Development, Network Assessment, and Budget Comparison. *AGU Advances* **4**, e2023AV000956 (2023).
6. R. B. Jackson, *et al.*, Increasing anthropogenic methane emissions arise equally from agricultural and fossil fuel sources. *Environ. Res. Lett.* **15**, 071002 (2020).
7. S. J. Sharp, *et al.*, Net Methane Production Predicted by Patch Characteristics in a Freshwater Wetland. *Journal of Geophysical Research: Biogeosciences* **129**, e2023JG007814 (2024).
8. J. Joiner, Y. Yoshida, Satellite-based reflectances capture large fraction of variability in global gross primary production (GPP) at weekly time scales. *Agricultural and Forest Meteorology* **291**, 108092 (2020).
9. H. Chu, *et al.*, AmeriFlux BASE data pipeline to support network growth and data sharing. *Sci Data* **10**, 614 (2023).
10. G. Pastorello, *et al.*, Observational Data Patterns for Time Series Data Quality Assessment in *2014 IEEE 10th International Conference on E-Science*, (2014), pp. 271–278.
11. Y. Rosseel, lavaan: An R Package for Structural Equation Modeling. *Journal of Statistical Software* **48**, 1–36 (2012).
12. S. K. McFeeters, The use of the Normalized Difference Water Index (NDWI) in the delineation of open water features. *International Journal of Remote Sensing* **17**, 1425–1432 (1996).
13. L. Pearlstine, A. Higer, M. Palaseanu, I. Fujisaki, F. Mazzotti, Spatially continuous interpolation of water stage and water depths using the Everglades Depth Estimation Network (EDEN). *IFAS CIR1521* (2007).
14. M. Palaseanu, L. Pearlstine, Estimation of water surface elevations for the Everglades, Florida. *Computers & Geosciences* **34**, 815–826 (2008).
15. P. A. Telis, Z. Xie, Z. Liu, Y. Li, P. Conrads, "The Everglades Depth Estimation Network (EDEN) surface-water model, version 2" (U.S. Geological Survey, 2015).
16. J. Volin, *et al.*, Validation of a Spatially Continuous EDEN Water-Surface Model for the Everglades, Florida. *Department of Natural Resources and the Environment Articles* (2008).
17. M. Belgiu, L. Drăguţ, Random forest in remote sensing: A review of applications and future directions. *ISPRS Journal of Photogrammetry and Remote Sensing* **114**, 24–31 (2016).
18. L. Breiman, Random Forests. *Machine Learning* **45**, 5–32 (2001).
19. National Hurricane Center, Tropical Cyclone Climatology, http://www.nhc.noaa.gov/climo/#hrhm.
20. D. Lagomasino, *et al.*, Storm surge and ponding explain mangrove dieback in southwest Florida following Hurricane Irma. *Nat Commun* **12**, 4003 (2021).
21. D. Lagomasino, *et al.*, Storm surge, not wind, caused mangrove dieback in southwest Florida following Hurricane Irma. (2020).
22. B. M. Kilgore, Fire Management in the National Parks: An Overview in *Proceedings of the Tall Timbers Fire Ecology Conference*, (1974), pp. 45–57.
23. D. D. Wade, J. J. Ewel, R. H. Hofstetter, *Fire in South Florida Ecosystems* (Southeastern Forest Experiment Station, 1980).
24. J. W. Jones, A. E. Hall, A. M. Foster, T. J. Smith, Wetland Fire Scar Monitoring and Analysis Using Archival Landsat Data for the Everglades. *fire ecol* **9**, 133–150 (2013).

25. C. Teske, M. K. Vanderhoof, T. J. Hawbaker, J. Noble, J. K. Hiers, Using the Landsat Burned Area Products to Derive Fire History Relevant for Fire Management and Conservation in the State of Florida, Southeastern USA. *Fire* **4**, 26 (2021).
26. J. Eidenshink, *et al.*, A Project for Monitoring Trends in Burn Severity. *fire ecol* **3**, 3–21 (2007).
27. J. Fuentes, AmeriFlux BASE US-Skr Shark River Slough (Tower SRS-6) Everglades, Ver. 1-1. AmeriFlux AMP. https://doi.org/10.17190/AMF/1246105. Deposited 2016.
28. S. L. Malone, *et al.*, Eddy Covariance Tower Data from Everglades Towers, Florida: 2004:2021. Environmental Data Initiative. Deposited 2022.
29. S. L. Malone, *et al.*, Inventory of Eddy Covariance Tower Data in AmeriFlux from Everglades Towers, Florida: 2004-ongoing. Environmental Data Initiative. Deposited 2024.
30. G. Starr, S. F. Oberbauer, AmeriFlux BASE US-Elm Everglades (long hydroperiod marsh), Ver. 4-1, AmeriFlux AMP. https://doi.org/10.17190/AMF/1246118. Deposited 2016.
31. S. L. Malone, T. G. Troxler, AmeriFlux BASE US-TaS Taylor Slough/Panhandle, Ver. 1-5, AmeriFlux AMP. https://doi.org/10.17190/AMF/2331383. Deposited 2024.
32. G. Starr, S. F. Oberbauer, AmeriFlux BASE US-EvM Everglades Saltwater intrusion marsh, Ver. 1-5, AmeriFlux AMP. https://doi.org/10.17190/AMF/2229155. Deposited 2023.
33. W. B. Shoemaker, F. E. Anderson, A. Booth, M. Sirianni, A. Daniels, Potential Accumulation of Soil Organic Matter from Carbon Cycling within Greater Everglades Cypress and Pine Forested Wetlands data: U.S. Geological Survey data release. https://doi.org/10.5066/P9GFKJCY. Deposited 2020.
34. W. B. Shoemaker, F. E. Anderson, M. J. Sirianni, A. Daniels, “Carbon Fluxes and Potential Soil Accumulation within Greater Everglades Cypress and Pine Forested Wetlands” in *Wetland Carbon and Environmental Management*, (American Geophysical Union (AGU), 2021), pp. 371–384.
